# Repeated pattern detection on fabric: A survey and novel approach

**DOI:** 10.1371/journal.pone.0340797

**Published:** 2026-02-05

**Authors:** Ebru Ayyurek, Matteo Marcuzzo, Alessandro Zangari, Lorenzo Giudice, Gianluca Bigaglia, Mara Pistellato, Andrea Albarelli, Andrea Gasparetto

**Affiliations:** 1 Ca’ Foscari Department of Environmental Sciences, Informatics and Statistics, Ca’ Foscari University of Venice, Mestre, VE, Italy; 2 Interconnected Nord-Est Innovation Ecosystem/Temporary Project Centre/Ca’ Foscari University of Venice, Venezia, VE, Italy; Kafkas University: Kafkas Universitesi, TÜRKIYE

## Abstract

Modern textile industries frequently apply patterns, such as brand logos or motifs, in near-regular arrangements to create visually appealing products. Consequently, the application of computer vision for pattern recognition is highly valuable for automating production chains and reducing waste. In this work, we address the challenging task of automatically detecting repeating patterns on fabric images, accounting for real-world complexities such as variable lighting and intentional pattern variance. We begin with an in-depth literature review on repeated pattern detection, highlighting current trends, organizing them into a hierarchy of sub-tasks, and discussing the novelty of each paper. Subsequently, we propose a novel method to solve our specific instance of this problem, focusing on detecting patterns with sub-pixel accuracy. We conduct extensive experiments to compare its performance against several baselines from the literature. Our method can be applied with high precision to real-world problems without requiring training data, instead using an automatic calibration procedure with limited human supervision. On a small synthetic dataset, our method detects repeated patterns with a 96% recall rate and an average alignment error of less than 0.5 pixels in just a few seconds, making it competitive with all tested baselines. Finally, we release our dataset and the code for its generation to encourage further research in this area.

## 1 Introduction

Applying repeated patterns or logos is a common practice in the textile and materials industries to personalize the products for a specific brand. The automatic identification of these patterns is therefore of great value, both during production (e.g., ensuring patterns are not improperly cut or cropped) and in later stages, such as authenticity verification or defect detection.

These repetitions are typically arranged with less precision than in standard mechanical manufacturing. A primary reason for this is the subtle, natural variations found in fabrics and fabric-based products. Because of this, traditional model-based inspection systems and strict, measurement-based tests may fail to recognize complex high-level motifs and low-level texture defects.

In this context, the automatic and precise detection of repeated patterns is of particular interest to the textile manufacturing industry [3]. Such automation is essential for ensuring quality control, replicating designs faithfully, and boosting production efficiency, as manual inspection is both time-consuming and error-prone [64,113,124]. An automated characterization framework can be highly beneficial, enabling strategies like zero-defect and zero-waste production. However, solving this task requires methodologies capable of tackling the unique challenges posed by the strict requirements of the industry.

Traditional Computer Vision (CV) methods have long been employed in this sector [1]. More recently, Deep Learning (DL) based approaches have also been utilized [2], though, as we will discuss, these can be limited by the practical constraints of manufacturing environments. Nevertheless, both CV and DL provide solid methodological baselines for developing a wide range of solutions, from micron-level fabric analysis to whole-cloth pattern detection. The literature contains many such examples, with applications proposed for the fashion, automotive, and textile design industries [3].

### 1.1 Fabric repeated pattern detection

This work addresses a specific industrial application: the detection of repeating near-regular patterns on fabric from high-resolution camera images. The task is of significant scientific and industrial relevance, as its unique constraints render many methods proposed in the literature unsuitable, as we will discuss in later sections.

We define this task as *fabric repeated pattern detection* (FRPD) to distinguish it from more general *repeated pattern detection* (RPD). In our specific use case, the patterns are company and brand logos, which may include both text and graphics. The goal is to assist human operators who manually cut the fabric. This process often leads to significant material waste when logos are improperly cut, which is a considerable issue at industrial production scales. In this context, our system aims to mitigate this by automatically pinpointing pattern centers to guide the operator during the cutting process.

The technical setup involves large fabric pieces that are manually placed on a flat surface. Pictures are then taken from one or more RGB cameras placed above it. Due to the large surface area, multiple images are captured and stitched together to form a single, high-resolution composite image. In our specific setup, the final image is composed of four individual captures.

**Fig 1:**
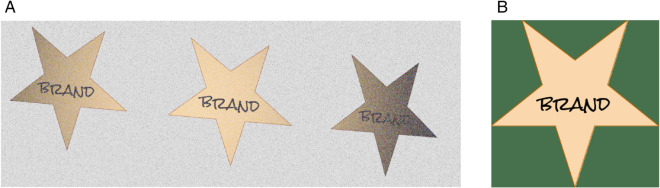
Example of distortions and light inconsistencies of a logo (b) rendered on fabric. (a) Aligned logos with added warp, shear, and noise. (b) Original logo.

As one might expect, this acquisition process introduces several challenges to accurate pattern detection. First, the stitched fabric image may contain subtle geometric distortions arising from camera perspective, lens specifics, and uneven fabric placement [103]. Furthermore, photometric inconsistencies are common, including pixel noise caused by non-uniform lighting across the different captured sections. The camera itself can also introduce optical distortions, particularly near the edges of each frame [119]. An exaggerated example illustrating these variations is shown in Fig 1.

A viable industrial solution for this task must satisfy several constraints:

- *Speed*: once calibrated, the method must deliver results in a matter of seconds;
- *Sub-pixel precision*: the method must locate pattern centers with a sub-pixel error;
- *Semi-supervision*: it must operate with minimal human input and generalize to new fabrics of the same type without extensive retraining.

This final constraint is critical because fully-supervised approaches are unsuitable for this task. In our target industrial setting, materials and logos change frequently, making it impractical to create and maintain a comprehensive labeled dataset for general usage. Moreover, as we will discuss, datasets with annotated examples for this specific problem are generally not available. Our proposed method, designed to meet these requirements, is described in Sect 4, and the experimental setup is detailed in Sect 5.

### 1.2 Contribution

In this work, we provide an extensive review of the literature on fabric pattern detection and address a specific industrial use case with a unique set of constraints. To the best of our knowledge, this is the first work to formalize and solve this particular problem, as we did not find any solution to this problem in the literature outside of standard CV methods. Thus, we develop a novel solution to this task and compare its performance against several baselines. Our approach involves an iterative sequence of template matching, registration, and super-resolution steps to improve detection quality, even under challenging conditions.

In summary, the contributions of this article are the following:

- We review existing works on RPD, outlining the main tasks related to this research topic;
- We showcase a novel method for the detection textile patterns, and benchmark its performance against other methods from the literature;
- We design our solution to operate under strict constraints, including speed, sub-pixel precision, and lack of training data;
- We release our sample of synthetic test images and the code to generate them, to facilitate future extensions and further research on this topic. Code for data generation and the dataset are available at https://github.com/ebruayyurek1/synthetic-fabric.

### 1.3 Structure of the article

This article is organized as follows. Sect 2 provides an analysis of the literature, giving an overview of the main sub-tasks and approaches related to and utilized in FRPD. In Sect 3, we survey existing research on RPD, focusing on FRPD. This section describes the relevant papers and organizes them into a taxonomy. Sect 4 introduces the proposed method and Sect 5 details the experimental setup, datasets, and metrics used to evaluate and compare our method to other baselines. We discuss our findings in Sect 5.6 and conclude this paper in Sect 6, which also describes possible directions for future research.

## 2 Background

In this section, we briefly introduce the most important classes of methods and techniques commonly employed to address FRPD tasks and discuss the state-of-the-art and the latest developments for each of them. The detection of repeated patterns is a broad task that typically involves multiple steps in a standard pipeline. Some of these steps have also been used in our proposed strategy, which will be detailed later.

### 2.1 Object detection

Object detection is an important task in CV, enabling the precise identification and localization of objects within images or video frames [4]. Traditional methods include the Haar Cascades [5], Template Matching [6], feature-based methods such as Histogram of Oriented Gradients (HOG) [7], Scale-Invariant Feature Transforms (SIFT) [8] and Speeded-Up Robust Features (SURF) [9], and traditional machine learning models like Deformable Part Models (DPM) [10].

With the rise of DL, more advanced, neural-based techniques have been developed. Some of the most notable methods include R-CNN, which integrates region proposals with Convolutional Neural Network (CNN) [11], as well as its later advancements Fast R-CNN [12] and Faster R-CNN [13]. Architectures like Single-Shot object Detection (SSD) [14] and You Only Look Once (YOLO) [15] focused on further reducing inference time to allow for real-time usage. Recent works have highlighted the fast progress of object detection approaches in the DL era. Notably, [16] provide a comprehensive survey of the recent achievements in this field brought about by DL techniques, with a comprehensive study including more than 300 articles. More recently, [17] have also provided an extensive examination of how object detection has evolved in the past few years.

Object detection methods have been widely used in the textile industry [18]. For instance, it has been used to improve quality control by identifying fabric defects, ensuring high-quality products, streamlining sorting and grading processes, and optimizing inventory management through automated recognition and counting of textile items [19]. This allows for a significant reduction in labor costs and errors while boosting productivity and fully automated systems.

### 2.2 Template matching

Because of its relevance in object detection literature and this work, this section will expand upon the basic yet fundamental technique of Template Matching (TM). First introduced by [20], TM algorithms work by trying to find the exact location of a template image in a larger image through a pixel-based measure of similarity. Templates are most commonly of rectangular shape, and the matching process slides the template over the larger image and compares pixel intensities at each position. Common similarity measures include the sum of squared differences (SSD) and (normalized) cross-correlation (NCC). At the end of the process, the target location is the area of the reference image with the highest similarity score [21]. In the context of object detection, a subsequent step of non-maximum suppression (NMS) [22] is commonly paired with template matching to refine bounding box predictions by removing lower-confidence predictions.

Template matching techniques are particularly relevant when accurate detection is of critical importance [23,24]. Although simple to implement and computationally straightforward in its base form, it has several limitations in its sensitivity to scale, rotation, and lighting conditions. Multi-scale- and multi-orientation-based approaches attempt to solve these issues, usually through the utilization of features such as SIFT [8] and SURF [9]. Though much more robust, the computation of these features incurs a notable computational overhead. In recent years, TM has been enhanced through hybrid integration with contemporary algorithms. For instance, [25] present a novel similarity measure between sets of objects which leverages statistical properties of mutual nearest neighbors, with their results proving to be useful for template matching “in the wild”. [26] propose an deep learning architecture that improves image registration by bypassing traditional feature matching and mapping template parts to their likely positions on a reference image. Two loss functions simplify alignment by matching specific points, reducing errors from unclear boundaries while a Gaussian kernel then smooths and refines the matching process for enhanced accuracy. Recently, [27] propose a TM method based on differentiable coarse-to-fine correspondence refinement to alleviate some of the problems faced by traditional TM algorithms. Lastly, [28] propose a fast TM algorithm based on NCC that uses a dynamic programming strategy in order to eliminate the disadvantages of high computational complexity and slow calculation speed.

Template matching is utilized in many textile industry applications, mainly related to pattern recognition, defect detection, printing, and quality control. For instance, identifying and aligning complex designs on fabric is a core step of automated sewing and printing [29]. In defect detection, template matching compares fabric images with defect-free templates, effectively identifying discrepancies like tears, stains, and weaving errors [30–32].

### 2.3 Image registration

Image registration is another important image processing technique that finds applications in several fields, including the textile and manufacturing industries [33]. The process of image registration is most commonly utilized in situations in which two or more images are captured at a scene at different times, from different viewpoints or different sensors. Image registration algorithms attempt to align the images such that geometrically corresponding points coincide. For example, [34] discuss various image registration methods used for aligning fabric patterns, detecting defects, and analyzing fabric structures accurately, thereby enhancing the efficiency and accuracy of fabric image analysis processes. [35] propose an alignment technique using matched important features to achieve precise alignment in image processing applications. The authors emphasize the critical role of accurate image alignment for various scientific applications, including quality assessment and defect detection.

DL is also highly effective for registration because of its ability to automatically learn complex image features necessary for alignment. For instance, [36] propose flexible DL designs for unsupervised affine and deformable image registration by training a CNN to to register pairs of unseen images in one shot. There are also several recent reviews that study registration and alignment methods and their evolution in recent years, such as the one by [37]. The study by [38] focuses on reviewing multi-modal image registration/matching, starting from handcrafted up to DL-based methods. Lastly, [39] first introduce a fundamental concept of image registration by following recent developments based on machine learning methods that have advanced to traditional methods.

Image registration methods usually rely on feature detection algorithms, whose features are then matched between the images to align. Features are usually points, edges, or distinct regions within the images that are to be used as reliable landmarks in their alignment. Common techniques for this purpose are the already mentioned SIFT, SURF, and Harris corner detection algorithms. After features are extracted, a feature-matching step is required, which involves matching corresponding image features. Correspondences between the images can be established using algorithms like nearest neighbor search to align the images, random sample consensus (RANSAC), iterative closest point (ICP) [37], and normalized cross-correlation [40].

It is worth noting that image registration techniques are also negatively affected by changes in illumination, scale, and rotation. Sophisticated methods have been developed to overcome these problems, such as mutual information-based registration and Fourier transform-based techniques [41]. Lastly, an important image registration technique is that of optical flow, which aims to estimate the motion between consecutive frames in a sequence of images. Optical flow algorithms compute the estimated displacement field difference between two images, efficiently mapping each pixel in one image to its corresponding pixel in the next [42].

Similarly to object detection approaches, the textile and manufacturing industry often utilizes image registration techniques in quality control processes [43]. Image registration helps in aligning images taken under different conditions or from different cameras, to ensure uniformity and accuracy in their analysis [44]. [45,46] discuss the use of image processing techniques for fabric analysis, including methods for texture and pattern identification with duplicate patterns. Though mostly limited to weave fabric pattern recognition, the approaches discussed are nonetheless relevant for FRPD.

### 2.4 Super-resolution

Super-resolution techniques, which aim to obtain high-resolution images from low-resolution ones, can be especially useful in the analysis of fine details required in the textile and manufacturing industry. Many conventional methods based on pixel interpolation and reconstruction exist. In short, interpolation techniques guess new pixel values by averaging the values of surrounding pixels to create smoother images, with examples including bi-linear and bi-cubic interpolations. Reconstruction-based methods, such as Iterative Back-Projection and sparse coding, rely on image priors and redundancy to infer the high-resolution details from low-resolution inputs by solving optimization problems [47–49].

In recent years, the state-of-the-art super-resolution approaches have increasingly leveraged the power of DL, utilizing large datasets and robust neural network architectures [50,51]. More specifically, CNNs proved effective in learning complex mappings between given low- and high-resolution images. A few examples can be found in SRCNN (Super-Resolution Convolutional Neural Network) [52] and SRGAN (Super-resolution Generative Adversarial Network), which set new benchmarks in this field [53]. We point to a recent overview by [54] for a description of key algorithms in the field of image super-resolution, including DL approaches based CNNs and GANs. There has been some work exploring the application of super-resolution in the textile industry, either for more accurate identification of defects or to generate higher quality patterns. For instance, [55] introduces a new framework to help non-professional users design their fabric images. Their approach combines object and template patterns to identify repetitive designs in fabric images and uses a Deep Image Prior (DIP) based super-resolution technique to produce higher-quality designs.

## 3 Literature review

This section details our comprehensive literature review. Each scientific study was evaluated in the context of pattern extraction and examined in detail. Papers are loosely grouped according to the base method utilized. Analyzed works are also summarized in Table 1, detailing the methodologies employed, the accessibility of the datasets, and the classification of the dataset types at the end of this section.

**Table 1:** High-level overview of the articles reviewed, categorized by their methodology, data source, general approach, and the public availability of their implementation.

Ref.	Methods Used	Dataset	Approach	Code
[61]	GLCM, Gabor wavelet, RDF	Fabric images	Feature	✗
[62]	LBP, SSIM	Fabric images	Feature	✗
[63]	Hough Transform, SVM	Fabric images	Classific./Statist.	✗
[64]	FCM	Fabric images (woven)	Classific./Statist.	✗
[66]	SVM, HMAX, GLCM, Radon Transform	Fabric images (woven)	Hybrid	✗
[67]	Improved KNN	Fabric images (yarn-dyed)	Classific./Statist.	✗
[68]	Spatial-domain integral projection	Fabric images (weave)	Feature	✗
[69]	SVM	Fabric images	Classific./Statist.	✗
[70]	GA, WFCM	Fabric images (embroidery)	Hybrid	✗
[71]	FCM, Hough Transform	Fabric images	Hybrid	✗
[83]	TM, SAD	Fabric images	Feature	✗
[46]	SURF	Fabric images	Feature	✗
[132]	Similarity based	Fabric images	Feature	✗
[74]	MRF model, BP	Non-fabric images	Segmentation	✗
[72]	KNN, MRF	PSU	Hybrid	✓
[102]	Cascade R-CNN	Fabric images	Deep Learning	✗
[75]	Wavelet transform, Sobel filter	Fabric images	Frequency	✗
[76]	Fourier transform, Euclidean distance	Fabric images (yarn-dyed)	Frequency	✗
[77]	SIFT, Hough transform	Fabric images (batik)	Feature	✗
[78]	LFS	Fabric images (woven)	Feature	✗
[79]	GHOG, Gabor filter	Fabric images	Frequency	✗
[81]	Radon signature, SIFT	CCNY	Hybrid	✓
[84]	Frequency domain methods	Fabric images	Frequency	✗
[85]	Wavelet transform	Fabric images (jacquard)	Frequency	✗
[86]	Fourier transform	Fabric images	Frequency	✗
[87]	Fourier transform, K-means, TM	Fabric images	Hybrid	✗
[88]	Haar wavelet transform, Elo rating	Fabric images (motif based)	Frequency	✗
[89]	DT-CWT, Gaussian filtering	Fabric images	Frequency	✗
[90]	Fourier transform, SIFT	Fabric images	Hybrid	✗
[115]	OCT imaging	Fabric images (woven)	Segmentation	✗
[117]	EMD, Mean shift segmentation	Fabric images	Segmentation	✗
[92]	Adaptive lattice segmentation, E-V method	Fabric images	Segmentation	✗
[118]	MRF, BP, SURF	Fabric images	Hybrid	✗
[120]	STA, SIFT, SVM	CCNY, UIUC	Hybrid	✓
[121]	Radon Transform, Wavelet Transform, HOG, FCM	Fabric images (hand-woven)	Hybrid	✗
[122]	Graph matching, Pixel wise methods	Fabric images	Segmentation	✗
[123]	DL and feature based	Fabric images (silk)	Hybrid	✗
[124]	Radon signatures, GLCM, KNN, SVM, DL	CCNY	Hybrid	✓
[125]	MTMSnet	Fabric images (weave)	Deep Learning	✗
[126]	Feature based methods (review)	CCNY	Feature	✓
[127]	TILT, HOG, FCM	Fabric images (woven)	Feature	✗
[128]	ATM, SAD	Fabric images	Feature	✗
[3]	FCM, Hough transform, GA (review)	Fabric images	Hybrid	✗
[93]	SIFT, K-means	Fabric images (jacquard)	Feature	✗
[94]	Multi-phase segmentation	Fabric images	Segmentation	✗
[95]	3D entropy	Fabric images	Segmentation	✗
[101]	CNN	Fabric images	Deep Learning	✗
[104]	Mask-RCNN	Elba and E-DTD	Deep Learning	✓
[105]	GLCM, NN	Fabric images (lombok songket)	Hybrid	✗
[106]	LBP, ANN	Fabric images (sarong)	Hybrid	✗
[107]	CNN	Fabric images	Deep Learning	✗
[108]	CNN, Hough voting	NRP, ECP facade [136]	Hybrid	✓
[109]	CNN, TM	Non-fabric	Hybrid	✗
[99]	CNN, TM	Non-fabric images	Hybrid	✓
[110]	CNN	Fabric images (woven)	Deep Learning	✗
[65]	CNN, LBP	Fabric images (woven)	Hybrid	✗
[111]	CNN	Fabric Images (FI), TILDA	Deep Learning	✓
[56]	BBs	Fabric images	Feature	✗
[57]	RBs	Fabric images	Feature	✗
[60]	Image decomposition	Fabric images	Segmentation	✗
[112]	HOG, PCA, LVQ	Non-fabric	Feature	✗
[113]	ANN, FCM	Fabric images (woven)	Hybrid	✗
[114]	ML methods (review)	CCNY	Classific./Statist.	✓
[96]	DL, Segmentation based method	Non-fabric images	Hybrid	✗
[129]	Shape alignment, DTM	PSU	Feature	✓
[82]	SIFT	Non-fabric	Feature	✓
[130]	HOG, FFT, TM	Fabric images	Frequency	✓
[131]	Feature based methods, SVM	CCNY	Hybrid	✓
[100]	Segmentation based method, Canny edge	Fabric images (batik)	Segmentation	✓
[133]	Fog computing, GLCM, DL	Fabric images	Hybrid	✗

*Note:* The abbreviations used in the **Approach** column correspond to the following method categories: **Classific./Statist.** = Classification/Statistical Methods, **Feature** = Feature-based Methods, **Frequency** = Frequency-based Methods, **Segmentation** = Segmentation-based Methods, **Deep Learning** = Deep Learning-based Methods, **Hybrid** = Hybrid Methods.

### 3.1 Search strategy for relevant articles

To ensure a comprehensive review of the literature on FRPD, a systematic search strategy was employed. The primary research question guiding this review was: “What are the most effective methods for FRPD in the literature?”.

Relevant databases, including mainly Scopus https://www.scopus.com, IEEE Xplore https://ieeexplore.ieee.org/Xplore, ACM Digital Library https://dl.acm.org, SpringerLink https://link.springer.com, and Google Scholar https://scholar.google.com, were selected for their extensive coverage of the fields of computer science and engineering. Key concepts identified from the research question included “fabric pattern”, “image extraction”. and “pattern recognition”. These concepts were combined into the following search string: *(“fabric pattern” OR “textile pattern” OR “fabric image” OR “textile image”) AND (“pattern extraction” OR “image extraction” OR “pattern recognition” OR “pattern recognition”)*. The initial search yielded 200 articles, which were screened for relevance by examining titles and abstracts. This process resulted in 150 articles selected for full-text review, of which relatively 120 were included in the final analysis based on their similarity in purpose to our study.

### 3.2 Taxonomy overview

**Fig 2:**
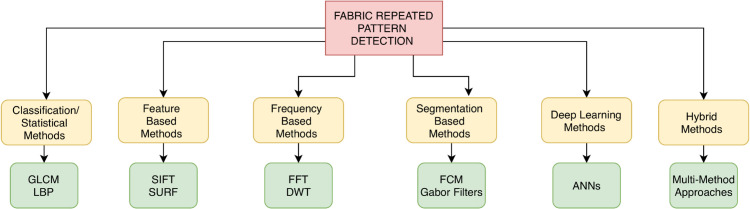
Taxonomy overview showing categories of FRDP methods. Lower boxes represent examples of the category.

The extensive literature presented in this section reveals a wide variety of methodologies applied to the extraction of fabric patterns. To facilitate a clearer understanding for scholars and practitioners, we have logically organized the reviewed methods in a taxonomy, summarized in Fig 2. Green boxes contain some example methods included in each general methods heading. This taxonomy classifies the reviewed studies based on core methodologies, application domains, technical approaches, and dataset utilization. We have also used this categorisation in Table 1 to classify each article according to the taxonomy and ensure clarity. By structuring the methods in this way, we aim to provide a clear and organized overview of how these approaches relate to and differ from one another, thereby offering useful insights into the FRPD field.

### 3.3 Classification and statistical methods

Classification methods in FRPD refer to algorithms used to identify, categorize, and analyze different patterns found in fabric images. Techniques such as gray-level co-occurrence matrices (GLCM), Gabor wavelet features, and structural similarity (SSIM) have been usually employed to detect and differentiate intricate patterns. Additionally, segmentation methods based on clustering algorithms and the Hough transform have been used to segment colors and identify repeating elements in fabric designs. In this section, we discuss classification methods relevant to fabric pattern detection.

[56] develop a fabric segmentation approach based on the financial concept of Bollinger Bands (BBs). During training, BBs are applied to rows and columns of the reference images for computing upper, middle, and lower bands using moving averages and standard deviations. Threshold values are then determined from these bands. In the test phase, the same BBs are applied to the test images, and deviations from the thresholds point out defective regions. The developed approach separates patterns effectively by catching their periodicity and failures. An extension of this work is proposed in [57], utilizing Regular Bands (RBs) instead. RBs compute Light Regular Bands and Dark Regular Bands for each line and column of the fabric image, which capture the periodicity of patterned textures. Threshold values are learned from defect-free images during training. The results from rows and columns ensure good segmentation and isolation of regular patterns with irregularities.

In [58], the authors employ a combination of advanced methods to extract and detect patterns (lattices) in fabric images. The process begins with the lattice extraction model proposed by [59], which recovers the translational lattice of the patterned texture and helps classify the symmetry group, identifying representative motifs. For each motif, they create a series of circular shift matrices to ensure that each pixel in one motif interacts with every pixel in another, mitigating slight misalignment and distortion. The method calculates the energy of moving subtraction by summing absolute pixel differences to quantify motif similarity.

[60] propose an image decomposition approach that divides images into two main components: a cartoon structure, which marks out where the specific items are, and a texture structure, used for capturing repetitive patterns. The optimization model in the process aims to maximize the correlation between the texture component of the test image and a reference defect-free image such that the decomposed texture is representative of periodic patterns from selected areas on reference. Through texture pattern isolation, the system efficiently captures patterns that repeat over time.

[61] discuss a method of defect detection using a combination of GLCM and Gabor wavelet features, along with Random Decision Forest (RDF) classifiers. GLCM and Gabor wavelets are utilized in the fabric image texture eradication step while RDF is utilized to classify between defect and defect-free images.

[62] segment fabric images with an adaptive strategy, based on the periodic distance of the fabric patterns, utilizing a sliding window to traverse the entire fabric sample and identify repetitive pattern blocks. Their work provides an improved local binary pattern (LBP) feature-based method to measure the correlation of repeated pattern blocks, which can be used to accurately recognize patterns and segment textured images. Structural similarity was selected in this article to identify the difference between image blocks, to reduce the computation cost of algorithms, and to improve the detection efficiency.

[63] describe a comprehensive system developed for visually impaired people that employs various methods to recognize fabric patterns and colors. Key techniques include the Hough Line Transform, which finds straight lines by transforming the image to polar coordinates and locating peaks at curve intersections. Secondly, the Canny edge detection algorithm finds the edges of objects in images, reducing noise, and using hysteresis to maintain edge tracking. Finally, for pattern recognition, the system employs a Support Vector Machines (SVM) classifier that categorizes clothing patterns based on features like color, texture, and shape. These methods ensure accurate detection and recognition of clothing patterns. Another related study from [64] describes a method for extracting the lattices (patterns) of woven fabrics. The fabric weave patterns are identified by analyzing the warp and weft floats using pixel gray-level cumulative values. The Fuzzy C-Means (FCM) algorithm is then employed to classify them, resulting in a black-and-white digital matrix representing the fabric weave patterns. For classification, a two-stage neural network is used, with the first stage classifying the weave patterns into three main categories (*i.e.*, plain, twill, satin), similarly to [65]. The second stage further classifies them into more fine-grained sub-categories. However, the reliance on specific initial conditions and preprocessing steps can affect the system’s robustness to varying conditions and limit its applicability to different types of fabric.

The study by [66] proposes the integration of an SVM classifier and HMAX (Hierarchical Model and X) bio-inspired computational model for pattern identification. These models are particularly effective in identifying and categorizing intricate textures due to their ability to process and analyze high-dimensional feature spaces. The fabric image is segmented into pixelated grains for detailed texture analysis. Texture features are extracted using mean values in multiple directions and the GLCM algorithm, capturing both local and global attributes. Radon Transforms project the image along various angles, highlighting repetitive and structural features, and generating a signature that reveals the periodicity and orientation of the woven patterns.

[67] propose a method for fabric texture recognition using a double-sided fusion of reflection and transmission images. The method utilizes gray projection, an improved k-nearest neighbors (KNN) algorithm, and K-means clustering for classification and color recognition. The proposed method improves the recognition accuracy of yarn-dyed fabrics, minimizing color and texture disturbances in fabric recognition.

The study by [68] focuses on weave fabric pattern detection. To detect the patterns in weave fabric images, a spatial-domain integral projection approach is applied. This technique involves calculating horizontal and vertical integral projections of the gray-scale fabric image, which helps in identifying the positions of interstices among the yarns by locating the local minima in these projections. The local minima are then used to find the warp and weft separation lines. The intersections of these lines are recognized as the crossed areas, which correspond to the repeated patterns in the fabric. This approach ensures accurate segmentation of the fabric into its repetitive patterns by detecting the interlacing areas where the yarns cross over each other.

[69] present a texture extraction technique aimed at identifying cloth patterns based on visual textures present in fabric images. They first extract features by using a Fast Texture Estimation Algorithm. Then, the extracted global and local features are combined to recognize patterns through an SVM. The method utilizes various texture measures to quantify and recognize patterns effectively. The technique excels in handling diverse visual patterns across various types of cloth, though its performance can degrade with highly complex or overlapping patterns. Nonetheless, it demonstrates high accuracy in controlled scenarios by successfully identifying distinct patterns in various fabric samples.

[70] extract the fabric lattice and its patterns using a combination of filtering techniques. First, they distinguish between repeated and non-repeated pattern embroidery images and use different methods to select regions of interest to reduce the computational load. For repeated pattern images, a genetic algorithm (GA) identifies smaller sub-images within the original image that share the same color components and spatial structures. For non-repeated pattern images, a discrete wavelet transform (DWT) is used to isolate low-frequency sub-images that retain important features while improving computational efficiency. The weighted fuzzy C-means (WFCM) method is then employed for color and region separation. Limitations include its reliance on input image quality and the assumptions of specific types of fabric.

[71] present a method for extracting and segmenting the repeated patterns of printed fabrics. A FCM clustering algorithm, coupled with a specific cluster-validity criterion, is used to segment the colors and identify pattern elements similarly to other works. Subsequently, the Hough transform is employed to establish repeated pattern segmentation by connecting the central points of identical pattern elements, which allows for precise measurement of the size of the pattern in both horizontal and vertical directions. However, the method’s reliance on precise initial image capture conditions can pose challenges in maintaining accuracy.

[72] present a method for automatically inferring the lattice structure of near-regular textures (NRTs) in real-world images. Though not specific to fabric images, the approach can easily be extended to them. The authors utilize the Patch-Match algorithm [73] to find KNN correspondences within an image. These KNN correspondences are used to recover an initial estimate of the 2D basis vectors and seed vertices of the texture lattice. The lattice is iteratively expanded by solving a Markov-Random-Field (MRF) optimization problem, discretized using the KNN, and optimized using a Particle Belief Propagation (BP) algorithm. The method, tested on a diverse NRT dataset, significantly improves texel detection and localization despite geometric and photometric distortions. However, it relies on precise initial conditions and complex MRF optimization. [74] present another method for tracking dynamic NRTs using a lattice-based MRF model. The authors define NRTs as geometric and photometric deformations from regular patterns that can move dynamically. The primary method involves using a lattice-based MRF model that captures the topological constraints among multiple textons (the basic units of texture patterns, also called texels) and an image observation model that performs local geometry and appearance variations. A tracking algorithm using BP and particle filtering maintains accuracy under various motions and lighting. However, it is computationally complex and requires initial texton detection.

### 3.4 Feature-based methods

While feature-based methods are often integrated into broader strategies, we describe in this section all works that focus on feature extraction and its application in FRPD. These approaches encompass a variety of techniques aimed at creating expressive and discriminative representations of patterns, as well as analyzing complex fabric designs. Key features utilized in these representations include color, texture, and geometric properties. Notable feature detection and matching algorithms applied to fabric images include the already mentioned SIFT, SURF, and HOG.

[75] describe a method for identifying and cataloging repeated patterns in fabric images for pattern retrieval. The image is processed using Wavelet transforms and the Sobel filter is used to detect repeated patterns using image gradients. Pattern descriptors are computed using color histograms, geometric features, and moment invariants, which guarantee invariance to rotation and translation. However, the method does not scale well to high-resolution images due to increased computational demands and storage needs. Furthermore, fabric tension during scanning can affect the accuracy of pattern segmentation.

[76] present an advanced method for the automatic identification and detection of color and weave patterns in yarn-dyed fabric, using local sequence images captured under reflected and transmitted light (LSRT). By applying the Fourier transform, the warp and weft yarns are pinpointed and segmented to identify weave points. To detect the woven pattern, incomplete weave pattern matrices from all sequence images are matched against the weave pattern database. The authors test eight LSRT images of each yarn-dyed fabric sample. Their proposed method, enhanced by error correction techniques, ensures precise and automated detection of fabric patterns.

[77] describe an advanced technique for identifying traditional cloth patterns (batik patterns) by leveraging SIFT for feature extraction and the Hough transform for improved precision. This method starts by detecting key points using SIFT. To handle the symmetrical and repetitive nature of Batik patterns, the Hough transform is applied to vote on the consistency of key points, removing mismatches and confirming correct matches. This combination ensures robust pattern recognition even with intricate Batik designs. The Hough transform plays a crucial role as a post-processing method by eliminating mismatched key points and retaining valid ones. This method improves accuracy with a lower error rate than original SIFT approaches. However, its computational intensity and sensitivity to noise and image quality may limit real-time use and affect recognition performance.

[78] propose a seven-characteristic method called Local Feature Similarity (LFS) to recognize weave pattern repeat automatically, focusing on twain, twill, and satin fabric. The task is that of identifying the number of wefts and warps in the rectangular area that represent a weave pattern repeated characteristic. LFS can be applied to the three primary weave patterns and their expansions (such as “warp rib”, “weft rib” and “panama”). The results proved the high degree of applicability and robustness of the LFS method. Compared with state-of-the-art methods mentioned in their article, the method has the highest recognition rate on the whole sample, as well as an average execution time of just a few milliseconds.

The work by [79] presents a method for defect detection in patterned fabric images (star, dot, and box patterned images) using a HOG-based method. To efficiently characterize the fabric texture feature, a novel second-order direction-aware feature descriptor is introduced, namely a combination of Gabor filters and HOG (GHOG). This technique captures gradient orientation features (*i.e.* texture-related), which are used to distinguish between defect-free and defected regions in patterned fabrics. The method shows high accuracy in detecting defects across various fabric patterns. The strengths include its effectiveness in handling complex patterns with high detection accuracy. However, the method may require extensive computational resources for processing high-resolution images.

Research by [80] proposes a method for extracting pattern features from clothing textures by fusing texture and color information. This approach integrates multiple features to provide a comprehensive analysis of the fabric, capturing intricate details in both texture and color. Discrete Fourier Transforms are used for template registration of garment texture patterns. The 3D garment texture pattern is adjusted using a two-dimensional function that fits the reference image to the offset between the reference point and the matching point. The Radon transform reorganizes the RGB components of the clothing texture pattern to extract features. Template pixels are used to obtain original image values for image template matching.

[81] use both local and global features of the pattern and colors in their system, which is used to precisely recognize 4 major patterns (plaid, striped, irregular, and pattern-less). The Radon signature identifies the principal orientation of the image to extract global features of clothing patterns. Statistical properties from wavelet sub-bands are utilized for this purpose. To extract local features, SIFT detectors are employed. Both local and global features are then integrated to recognize complex clothing patterns.

[82] propose to detect repeated patterns of visual words in images. They build a feature graph consisting of local features at various locations and scales with SIFT descriptors. Detection is performed by checking that features are connected by an edge when close and with similar scale and appearance.

[83] detail a method for FRPD using template matching techniques. The approach starts by selecting a template from the input image and separately extracting the RGB color components from both the template and the input image. The Sum of Absolute Differences (SAD) is used to compare pixels, and approximation errors are calculated for each pixel. The mean and standard deviation of RGB errors are computed, summed, and squared to highlight differences. Blocks are ranked by similarity scores, with the lowest indicating the most similar patterns. Euclidean distance ensures unique pattern detection. Challenges include oriented or skewed patterns, size deviations, and missing parts, affecting accuracy and specific fabric types.

[46] focus on fabric image registration and color difference detection based on cyclic elements. Their work uses the SURF algorithm to segment fabric images according to pattern cycles, utilizing the working characteristics of fabric inspection machines. Position information of the cyclic elements is added through the detection of SURF features. Feature points with the same cyclic element positions are matched across images to perform registration.

### 3.5 Frequency domain methods

Frequency-based methods are frequently used in literature for fabric pattern recognition. Methods such as FFT and DWT provide a powerful way to analyze and interpret the periodic and repetitive structures found in textiles. The core idea of these approaches is to transform spatial domain information into the frequency domain, thus allowing for the identification of patterns [84].

[85] focus on fabric defect detection and apply different methods to extract patterns from images of Jacquard fabric. In one of these, a DWT is applied to decompose the main image into sub-images or patterns. In this process, fourth-level decomposition is employed to highlight key details that represent the patterns. These sub-images are then subjected to thresholding to pinpoint and extract regions corresponding to the fabric pattern. The selected sub-images undergo further enhancement, allowing for the effective isolation of the desired patterns.

[86] aim to find fabric defects in textured surfaced images by developing a Phase Only Transform approach. Fourier transforms are used to retrieve the regularity of the images by analyzing repeated peaks in the signal. Instead of attempting to detect peaks in the Fourier transform of the input images, this method eliminates all regularities of various sizes and patterns by normalizing the Fourier transform by its magnitude. This process retains only the phase information while removing all regular patterns at all scales. An example of a frequency-based FRPD study is proposed by [87]. The study aims to extract the repeated fabric patterns from printed images in a fully automatic way. The authors use the Fourier transform, applying it to the preprocessed image horizontally and vertically. The repetition in the spatial domain is thus extracted, and the frequencies are clustered using K-means to detect the repeating pattern. TM is then used to pinpoint the patterns on the original image.

In [88], a pipeline for defect detection on patterned fabric images using the Elo rating method is proposed. After preprocessing to improve image contrast, a level 2 Haar wavelet transform reduces the image complexity while maintaining crucial pattern details. To extract the texture motif, candidate partitions are selected from these wavelet-transformed images. These are then matched on the original image, computing an Elo score matrix that captures the similarity between each patch and the golden partition. Hence, in this system, the Elo score defines the entity of the defect and achieves good results on their task-specific dataset.

[89] introduce a FRPD technique based on the dual-tree complex wavelet transform (DT-CWT) combined with Gaussian filtering for fabric image recognition. The DT-CWT approach effectively handles texture variations and reduces noise, making it robust to different fabric patterns. The computational complexity of the method is a notable weakness, requiring significant processing power and time. Despite this, the technique has proven to be highly robust, accurately extracting and recognizing fabric patterns in various experimental setups.

[90] explore the identification of repetitive elements and patterns through local features, using corner detection to identify potential repetitive elements. The authors use techniques from a previous study on pattern detection [91], and focus on the retrieval of similar images based on the repeated pattern. They address the inherent shift ambiguity in the repeated patterns by proposing shift-invariant descriptors based on the magnitude of the Fourier transform and color histograms of the detected elements. The Fourier transform captures frequency characteristics, making the descriptor shift-invariant, while color histograms enhance robustness to appearance changes. Local features are clustered and matched across views using this descriptor. The match score is based on tile, color, and lattice size similarity, producing a fronto-parallel view for verifying repetitive patterns across images.

### 3.6 Segmentation-based methods

In the case of fabric pattern recognition, segmentation techniques are considered as one of the base methods in the automation of identification and classification processes of complex patterns in textiles. These methods segment the distinct pattern elements of fabrics from their background, enabling detection and further analysis more precisely. In the following, we will discuss articles in the literature that use segmentation-based methods to perform fabric pattern recognition.

A review paper from [3] deals with segmented printed fabric patterns used in quality control for the textile industry. Among highlighted methods stand the FCM clustering algorithm, which is effective for color segmentation and segmenting patterns, and the Hough transform, which assists in the identification of repeated patterns by linking central points of the same elements. Further, the authors highlight how GA can be applied to aid in the segmentation to enable the detection of sub-regions with similar color distribution. Such methods can increase the precision and speed in detection, although the quality of the input image and preprocessing affects the efficiency of these techniques.

[92] outline a novel fabric defect detection approach based on patterned fabric images. To detect the repeated structures they propose an adaptive lattice segmentation method that extracts lattices representing the periodic structures. They learn the ideal pattern size for each segment using a customized loss function.

[93] presented a novel segmentation algorithm for Jacquard fabric with motif incorporated into the construction of the weave. Their method is based on multi-view image fusion and addresses challenges in accurately reflecting fabric surface information and facilitating subsequent processing. The SIFT method is used for image registration, paired with wavelet decomposition to extract texture features. K-means clustering is then used to segment the fabric images, showcasing excellent performance on their dataset.

[94] propose a novel method for fabric pattern recognition and recoloring. The main focus of the study is to segment the color pattern from the background of patterned images, applying a multi-phase segmentation method to extract the motif pattern from the fabric image.

[95] introduce a segmentation approach to distinguish fabric patterns in images. The authors design an improved fruit fly optimization algorithm to determine the threshold value for image segmentation. Segmentation is then achieved through a 3D entropy approach.

[96] propose an unsupervised segmentation approach based on differentiable feature clustering to extract the repeated elements in images. The segmentation approach is based on the work by [97], and is used to extract the design. The background mask is extracted first, and then pixel values are compared to the background to find the foreground elements. Finally, elements are extracted following the work by [98] and their previous work that we mention in our literature review [99], additionally using Canny edge detection to enhance contours in case not all the elements are extracted.

[100] use diverse preprocessing techniques to accurately capture the characteristics of the image patterns. Textile units are isolated and filtered to detect essential elements, and are transformed into binary images for subsequent boundary extraction. The Canny algorithm is employed to extract the overall contour of the batik fabric pattern and its elements, resulting in contours that can be read and edited independently. Experimental results using various batik fabric images and segmentation methods highlight the effectiveness of their approach.

### 3.7 Deep learning methods

As DL methods continue to advance across various fields, their impact on the textile industry has become increasingly significant. In particular, CNNs offer sophisticated techniques for identifying complex textile patterns. This section examines numerous studies that have utilized deep-learning techniques to improve fabric pattern recognition.

[101] propose a CNN algorithm with a spatial pyramid pooling module to detect the complex and simple periodic fabric pattern detection. Different from other CNN-based methods, the spatial pyramid pooling module is used to learn multi-scale features and extract rich feature information. The authors introduced a classification network for printed fabrics, employing transfer learning to train the CNN. The convolution layer of the trained network is used as a feature extractor, and the activation peaks are instead used to successfully segment the pattern primitives.

[102] detect defects in fabrics using a combination of advanced image processing techniques and machine learning algorithms. The authors employ a Cascade R-CNN framework enhanced with Switchable Atrous Convolution layers to improve the feature extraction capabilities of ResNet-50 and modify the Feature Pyramid Network for better detection accuracy of small defects. The method used to extract repeated patterns in fabric involves a block recognition algorithm, where high-resolution images are divided into smaller blocks during both training and inference phases. These blocks are then processed, and the detection results are merged using a detection box merging algorithm, which employs NMS to eliminate redundant detections.

In [104], the authors present a fine-grained attribute-based framework to represent and classify identifiable elements in fabric textures. First, a Mask-RCNN is used to detect individual texels and assign them attributes related to their shape, color, orientation, and size. The texels are then grouped, and layout attributes are used to describe the spatial patterns of texels. The authors also produce and publish two datasets of labeled textures, namely the Elba and Element-based DTD (E-DTD) datasets.

[105] employ a combination of GLCMs and NNs to extract and identify Lombok songket fabric patterns. A GLCM method is used to extract texture features by calculating parameters such as mean, variance, energy, entropy, contrast, dissimilarity, and homogeneity. These extracted features are fed into the NN, which is trained to classify the motifs. The paper highlights the robustness of combining GLCM for feature extraction with NNs for classification, achieving a high recognition rate. However, a notable drawback is the system’s reduced performance with motifs of varying colors and angles, suggesting a need for improvements to handle these variations effectively.

[106] explores using LBP to extract texture features from grayscale images. These features are then refined through Correlation-Based Feature Selection (CFS) and Principal Component Analysis (PCA) to reduce dimensionality and improve discriminative power. The refined features are input into a NN for classification. Because this method depends on high-quality images, it may be vulnerable to changes in lighting and angles during image capture, which could impact feature extraction and classification.

[107] propose a fabric pattern recognition approach that utilizes NN in combination with fabric properties. The quantitative analysis of impact resilience and drape attribute these properties to two factors: fabric properties derived from parametric modeling and those obtained through geometric measurement. The image measurement data is normalized and analyzed using polynomial regression. A CNN used to adaptively extract features based on image characteristics The fabric recognition model developed by the authors is designed to learn the parameters of these two fabric attributes, thereby enabling automatic fabric recognition.

[108] introduce a method for detecting repeated patterns on a grid within individual images through a multi-level feature extraction and feature clustering based on a CNN architecture. This approach utilizes the responses from pre-trained deep CNN filters across various layers, capturing features at different levels and scales. Displacement vectors for all filters and layers cast votes into the displacement vector Hough voting space. Finally, an implicit pattern model is built to detect the instances of the pattern that models the grid. This makes the method highly robust, handling changes in visual appearance caused by occlusions, lighting, or background clutter. When compared to keypoint-based methods, the method has significant recall improvements, despite some losses in precision, while also struggling with highly irregular or noisy patterns. Subsequent studies by [99] and [109] continue this line of work, proposing faster alternatives that work in broader situations such as ones with different-sized patterns and differently aligned patterns.

[109] introduce a novel pipeline for identifying minimal repeatable patterns in both fabric and non-fabric images. By using the feature space of a CNN, they aim to find the size of the minimal repeating pattern as well as a list of repeated tiles. Through the combination of template matching and Gaussian blending methods, a tiled texture of the same size as the input image is created. Finally, by using deep perceptual loss between the tiled image and the input image, the optimal tile (that can best represent the original image) is discovered.

[99] showcase a method for identifying repeated fabric patterns by combining CNNs with template matching. First, the method selects the most activated filters from pre-trained CNNs that capture repetitive patterns. The peaks are extracted using a NMS algorithm, and a Hough-like voting method calculates the consistent displacement vector, indicating the size of the repeated pattern. Template matching is then used to fine-tune this displacement vector for greater accuracy. The method may encounter difficulties with patterns that have significant visual irregularities or those not effectively captured by the selected filters, though it can be effective in complex and non-uniform designs.

[110] propose an automated and real-time fabric weave pattern recognition and classification system using a pre-trained CNN. Features such as fusion materials, shape, color, and texture are extracted using GLCMs, Gabor filters, and Random transforms. The combined pipeline showcases higher detection accuracy.

[65] focus on woven fabric pattern recognition and classification using deep CNNs, emphasizing the use of LBP and GLCM for feature extraction. A significant distinguishing factor of this work is the utilization of large amounts of datasets, which contain images with varying acquisition conditions such as uneven lighting and rotational variations.

[111] introduce a method for detecting fabric defects using a CNN combined with repeated pattern analysis. The approach involves a CNN-based detector that leverages the network’s feature activations to identify repeating patterns in the fabric to capture high-level contextual semantics and periodic elements. To enhance the model’s capabilities, the authors use a semi-supervised learning scheme that incorporates knowledge of periodic patterns into the network. This allows the method to operate independently of additional pre-calculations, maintaining both detection speed and network efficiency. However, the reliance on pre-computed labels for initial training could be seen as a limitation due to the extra computational overhead.

[112] present a method for recognizing patterns in Balinese carving motifs using Learning Vector Quantization (LVQ). Their research focuses on feature extraction and classification of Bali carving patterns using HOG and PCA techniques trained with LVQ. They compare the performance of HOG and PCA, finding that HOG performs better. However, the method may face challenges due to variations in carving styles and the quality of input images, which can affect recognition accuracy.

[113] propose a method that first extracts texture features using a white-black co-occurrence matrix, and then applies an ANN to automatically recognize woven fabric patterns. The authors also use FCM to classify floats of woven fabric images. The ANN is trained to learn the complex relationships between the texture features and the corresponding pattern classes, leading to high classification accuracy while also being demanding in terms of computational overhead.

In their review, [114] focus on neural network-based fabric image pattern recognition for visually impaired people. The authors use wavelet sub-bands to extract global features of clothing patterns, combining them with local features that are obtained from SIFT features to recognize complex clothing patterns.

### 3.8 Hybrid methods

Hybrid methods in FRPD combine multiple techniques to leverage their strengths, aiming to be more accurate and robust overall systems. These methods integrate traditional image processing, statistical, and sometimes ML techniques to address the complexities and variations inherent to fabric textures. This section explores several studies that implement hybrid approaches.

[115] proposes an approach to detect woven fabric weave patterns automatically by using spectral domain optical coherence tomography (OCT) imaging technique. OCT is a non-invasive, high-resolution imaging technique that creates cross-sectional images of an object, that also has been used in the textile industry [116]. To detect the repeated pattern a scanning process is repeated on the OCT signals until periodicity of the signal and the weave repeat can be identified, and iterated several times. This signal captures the reflection characteristics of the surface it interacts with, thereby revealing details about the weave pattern. Since the warp and weft regions produce distinct OCT signals, the scan profile enables the identification of the weave pattern.

[117] propose a hybrid combination of two-dimensional empirical mode decomposition (EMD) with texture-level Mean shift segmentation for fabric pattern recognition. The 2D EMD is initially employed to decompose the signal into its intrinsic mode functions. Notably, the first intrinsic mode captures multiple regular patterns present in the underlying fabric image, which is then utilized in an iterative clustering algorithm based on mean shift. The algorithm demonstrated good performance in clustering the diverse patterns inherent in textile images in a fully unsupervised manner.

[118] propose a method for fabric image color recognition based on pattern-driven fabric images using a multi-phase approach. The process starts with motif unit detection, identifying rotational and translational symmetries in the fabric image through improved corner detection, and mean-shift clustering. MRFs are used to model corner feature distribution of the repeating quadrilateral elements in the samples. A Confidence Belief Propagation algorithm is used to detect the repeated units and evaluate their rotation and distortion. The classification of color patterns is carried out by using a TM-based approach. The SURF algorithm is used to match tracking feature points between the selected target and potential candidates within the motif image.

[120] merge broad and detailed feature extraction methods to identify and interpret fabric patterns and fabric color recognition to help visually impaired people. Firstly, they use a Radon Signature descriptor to determine the main direction of an image, effectively capturing the overall orientation of patterns like plaid and stripes. Next, Statistical Texture Analysis (STA) is used to break down the image into several sub-bands at different scales, also extracting important statistical features. SIFT is also used to extract local structural features; combined, the features form a bag-of-words model. The combination of these global and local features is the input of an SVM classifier. The authors report that a combination of SIFT and Radon signature methods yielded the best performance.

[121] offer a comprehensive analysis of various techniques for recognizing hand-woven fabric motifs, emphasizing the importance of a multi-step approach that includes image preprocessing, feature extraction, feature selection, and ML. The study highlights the benefits of using a combination of advanced techniques for high accuracy in motif recognition. The combinations tried include Adaptive Wiener Filtering and Histogram Equalization, Radon and Wavelet Transform, Transform Invariant Low-Rank Textures (TILT), HOG, and FCM. Additionally, the study provides insights into the challenges presented by the variability in datasets found in the literature.

[122] present a comprehensive methodology for automating the quality inspection of printed fabric patterns in the textile industry by using graph matching and the pixel calculation algorithms. The system starts with automatic image registration to capture fabric pattern characteristics, followed by fast image registration using graph matching to identify shapes within the selected area. The images are further processed using various techniques, such as edge detection with first and second-order derivatives, and histogram analysis for initial segmentation. While the proposed method is well-suited for real-world textile manufacturing applications, its effectiveness can be influenced by the quality of the initial image capture.

[123] explore the effectiveness of different feature extraction techniques for recognizing silk fabric patterns. The study focuses on two primary methods based on texture and local feature descriptors. For texture features, a LBP method is used to analyze local structures in the fabric image, while local feature descriptors use a combination of HOG and SIFT to extract gradient orientations and key points, respectively. The extracted features are then fed into KNN and SVM classifiers. The results obtained by the authors indicate that the LBP method combined with KNN or SVM perform better than other techniques, including DL architectures like LeNet and AlexNet. Though limited to silk fabric, the study highlights the robustness and efficiency of combining LBP with traditional classifiers for pattern recognition, while also indicating that DL models may struggle because of their requirement for more extensive training data to be effective.

[124] propose an automatic cloth pattern recognition technique designed to classify patterns using a combination of traditional image processing, ML, and DL hybrid techniques, specifically for visually impaired individuals. To recognize fabric image patterns, both global and local features are extracted. The article employs Radon signatures and the GLCM for feature extraction. For pattern recognition, ML algorithms such as KNN and SVM, as well as DL networks, are utilized. The authors indicate that DL approaches achieve higher overall accuracy due to their ability to automatically extract relevant features.

In [125], several ANNs for pattern prediction are analyzed and discussed, focusing on particular weave fabric images. The authors discuss the challenges of recognizing woven fabric, while also proposing a multi-task and multi-scale convolutional neural network (MTMSnet).

[126] propose a method for recognizing fabric patterns and colors to assist visually impaired individuals. The authors present several approaches for this purpose. The first method identifies five different clothing patterns (vertical and horizontal stripes, checker, plain, and other) using Fourier transforms and Sobel operators. The second method employs feature-based techniques to extract global and local features using a Radon signature, STA, and SIFT. The final method utilizes Recurrence Quantification Analysis (RQA) as a local feature extractor, in combination with STA and SIFT. The paper reports and discusses the results of each method.

[127] present an effective method for identifying woven fabric patterns using TILT and HOG. The proposed method addresses issues like image skew during fabric image acquisition by applying TILT for correction. Subsequently, the yarn floats are localized and segmented using a 2D spatial-domain gray projection to differentiate between weft and warp yarns. HOG is then utilized to extract distinctive texture features. FCM is employed to classify the yarn floats, enabling the recognition of woven fabric patterns such as plain, twill, and satin.

[128] present a scaling and rotation invariant method for extracting repeated fabric patterns using adaptive template matching (ATM). The technique starts by selecting a template image automatically based on areas with the highest edge density (extracted using a Canny edge detector). The SAD is used as a metric to find matches in the original image. The minimum envelope border of the pattern, typically a parallelogram, is identified by analyzing adjacent matched templates. The smallest parallelogram formed by non-collinear displacement vectors is selected as the periodic pattern primitive that aligns closely with human perception while minimizing information loss.

[129] propose a simple approach to extract the repetitive pattern in near regular textured images. They accomplish the detection by shape alignment and deformed template matching (DTM) for the local patches of the texture. In their study, they define a transformed patch space to perform the detection. They also assert that this pipeline is adequate for application to fabric images.

In the recent work of [130], the authors propose an adjacent key-point localization framework to automate FRPD on printed fabric. First, they segment the images into sub-patterns and select those with high connectivity and information entropy (the seeds). They extract the HOG and FFT features from both the original image and the seed sub-patterns. TM is then used to detect similar sub-patterns, followed by NMS.

[131] discuss various techniques for extracting features from fabric texture patterns to facilitate classification, namely SIFT, the Radon Transform, and other statistical feature extraction methods. While SIFT and the Radon transform are used because of their robustness and invariance properties, statistical features such as energy, entropy, and variance are also calculated to capture the global characteristics of the texture patterns. The extracted features from the previous methods are then classified using a SVM with an RBF kernel.

[132] introduce a method using similarity spaces to automatically identify the consecutiveness of textile patterns. The authors decompose the pattern consecutiveness into two factors: repeated angles and unit spans. The pattern images are sliced at various angles and spans, and the similarity degree of these slices is calculated to construct a similarity space. Peaks in this space indicate potential repeated angles and unit spans. The angles are analyzed in similarity space to differentiate between four-consecutive and two-consecutive patterns. Additionally, peak significance is employed to distinguish sharp peaks from weak ones, such as to identify consecutive patterns. While this method is effective for recognizing repeated angles, its simplicity can hinder performance when applied to images with significant defects or variations in quality.

Differently from conventional fabric analysis that involve centralized data processing [133] propose an alternative a fog computing which is a computing architecture that distributing handling information and positioning computation nearer to the data source by aiming the faster and effective way, particularly when dealing with extensive datasets and intricate fabric designs. GLCM methods are also utilized to extract features, while DL techniques are used to classify the patterns into dotted, floral, solid, and striped categories.

### 3.9 Gaps in literature

Despite the advances in detecting fabric image patterns, our literature review indicates that there are still knowledge gaps that require further research:

- **Diversity of Fabric Types and Patterns**: Many studies focus on specific types of fabrics, namely woven type fabric (the most common), satin, and Jacquard. However, there is a lack of methods that are designed to effectively handle a variety of fabric types and complex, diverse patterns. Future research could benefit from developing more generalized approaches that are not limited to only specific fabric types or patterns. This issue was also discussed by [134].
- **Lack of publicly available fabric datasets**: There are currently very few datasets that can serve as public benchmarks, which may be attributed to the overspecialization of most studies (and datasets) to highly specific use cases. Future research should focus on establishing a public fabric image database that encompasses a diverse range of fabric patterns and structures, thereby facilitating the development of these algorithms [99].
- **Robustness to variations and defects**: Many existing methods struggle with variations in lighting, rotation, and fabric defects. There is a need for more robust algorithms that can accurately recognize patterns under diverse and less controlled conditions [135].
- **Reproducibility and accessibility of methods**: as mentioned by other authors [87], there is a notable lack of public implementations and solutions for fabric-related CV applications. We believe this issue arises from the prevalence of industrial contributions, which often cannot be publicly shared. Nevertheless, fostering more open research in this area could enhance accessibility and contribute to the advancement of the field.

Bridging the identified knowledge gaps and pursuing future research directions will facilitate advancements in FRPD, ultimately resulting in increased applications within the pattern recognition community.

### 3.10 Fabric image datasets

As mentioned, there is a scarcity of publicly available datasets for FRPD. Many studies develop custom datasets, but these are seldom made publicly accessible. Table 2 lists and describes the public datasets that have been frequently utilized in the literature.

**Table 2:** Publicly available datasets found in the literature.

Name	Size	Description
ElBa [104]	70 000	Synthetic geometric shapes on fabric textures
RPD [137]	841	774 scanned fabric images and 67 computer-generated
NRP [138]	> 50	Near regular textured images
PSU Near Regular Texture Database [72]	> 1 000	Fabric, non-fabric

## 4 Proposed method

In this section, we introduce our novel pipeline designed for the detection of an arbitrary number of repeated patterns within an image. Unlike many methods found in the literature, our approach does not require lengthy training on extensive datasets. We do this to ensure our method can be used when no annotated data is available, and with only limited input from the operators, as explained in Sect 1.1.

### 4.1 Operational workflow

Conceptually, our method is designed around a two-stage workflow to assist human operators in efficiently locating logos on fabric for precise material cutting. The two stages are a one-time *calibration* phase and a real-time *inference* phase.

**Fig 3:**
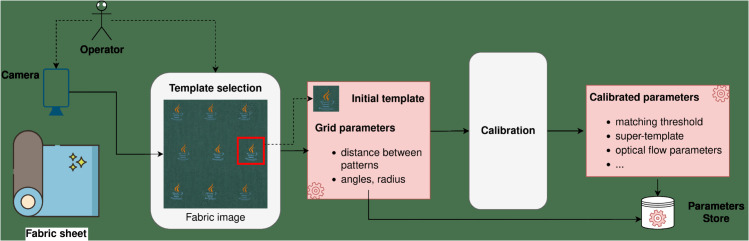
A schematic of the one-time calibration phase, where operator input is used to generate an optimized super-template and configuration.

The process begins with the *calibration* phase when a new material is introduced (Fig 3). Using a GUI-based tool, an operator identifies a representative logo (the *initial template*) and two of its immediate neighbors, a step we call *template selection*. This minimal input is sufficient to estimate the periodicity and orientation of the pattern grid. Following this manual step, an automated procedure optimizes internal parameters, such as cross-correlation thresholds, noise sensitivity and produces a high-resolution version of the selected template, which we term the *super-template*. Once complete, this configuration is saved, “registering” the material for future use.

**Fig 4:**
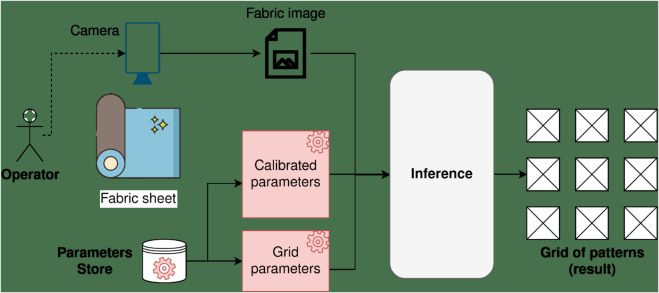
The real-time inference phase, where the saved configuration is used to quickly detect all patterns.

The inference phase (Fig 4) is the standard operational mode. For a previously registered material, the system loads the saved configuration and applies a streamlined detection pipeline to rapidly and accurately identify all pattern instances. These operational phases are built upon a shared set of core technical components, which we detail next.

### 4.2 Core pipeline components

**Fig 5:**
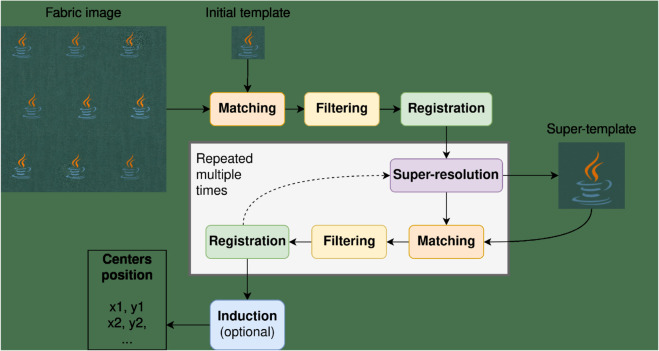
The complete pipeline for FRPD. The *super-resolution, matching, filtering* and *alignment* steps can be iterated to refine the detection and generate a high-quality super-template.

Our pipeline, illustrated in Fig 5, consists of five sequential building blocks. This sequence can be iterated multiple times to refine detection accuracy. The inputs to our pipeline are the fabric image, typically in high resolution, the *initial template*, and the *grid parameters*.

#### 4.2.1 Matching.

We use TM to identify candidate locations for repeated patterns. This is achieved by optimizing the NCC between a template and the source image (Eq 1). To focus matching on the pattern itself and ignore background texture, a circular binary mask is applied to the template.

$$R(x,y)=\frac{\sum_{x^{\prime },y^{\prime }}(T^{\prime }(x^{\prime },y^{\prime })\cdot I^{\prime }(x+x^{\prime },y+y^{\prime }))}{\sqrt{\sum_{x^{\prime },y^{\prime }}T^{\prime }(x^{\prime },y^{\prime }{)}^{2}\cdot \sum_{x^{\prime },y^{\prime }}I^{\prime }(x+x^{\prime },y+y^{\prime }{)}^{2}}}$$ (1)

In the equation, *x*,*y* represent the pixel coordinates of the top-left corner of the sliding window in the source image. The terms $\left(x^{\prime },y^{\prime }\right)$ are the local coordinates within the template, and the summation is performed only for the pixels where the circular mask is non-zero. $T^{\prime }$ and $I^{\prime }$ are the zero-mean normalized template and image patch, respectively, where the means are also calculated using only the masked pixels. Consequently, the numerator represents the dot product of the masked $T^{\prime }$ and each patch in $I^{\prime }$, and the entire expression is an indicator of their similarity. The resulting coefficient *R*(*x*,*y*) ranges from -1 (perfect anti-correlation) to +1 (perfect correlation), a similarity score which is robust to linear variations in brightness and contrast. In our pipeline, this step is applied twice. First, an initial *coarse* matching is performed using the template selected by the operator. After the super-resolution step creates an enhanced super-template, a second, *fine* matching is conducted between the fabric image and this higher-quality template to achieve more precise localization.

#### 4.2.2 Filtering.

The *filtering* step is applied immediately after matching to prune noisy candidates. This process analyzes each candidate patch and classifies pixels as outliers if their values deviate significantly from the mean or median. The two strategies did not differ much in our tests, hence we preferred the one based on mean and standard deviation since it operates slightly faster. Subsequently, candidates with an excessive number of outlier pixels are removed.

#### 4.2.3 Registration.

In the *registration* phase, we determine the affine transformation required to align each candidate with a reference template. This is achieved through a two-stage process that combines phase correlation with dense optical flow, as outlined in Algorithm 1.

First, we estimate the coarse translational offset between the reference and each candidate using *phase cross-correlation*. This method has been introduced in [139] and relies on a frequency-domain representation of the data. It establishes a relation between two signals that only differ by a translation and the phase shift of their Fourier transforms. After computing the phase difference of the two signals and the inverse Fourier transform, the resulting peak represents the required translational offset. Second, to account for more complex geometric distortions such as rotation and scaling, this initial alignment is refined using *dense optical flow*. We opted for the Dense Inverse Search (DIS) method [140], which offers a faster yet reliable alternative to traditional methods like the Farneback method [141]. Our empirical findings suggest that while the choice of this method had minimal impact on the overall results, DIS has the advantage of being consistently faster [142]. The resulting flow field, which contains a motion vector for every pixel, is used to estimate the refining affine transformation matrix. The final transformation for each candidate is then the composition of the initial translation and this subsequent affine transformation.

**Algorithm 1 Candidate registration.**

**Require:** Grayscale reference template *T*

**Require:** Grayscale candidate image *C*

**Ensure:** Affine transformation matrix *A*_*final*_

1: **function** REGISTERCANDIDATE(*T*,*C*)

2:   $\left(y_{shift},x_{shift})\leftarrow \text{PhaseCorrelate}(T,C\right)$    ▷ Coarse translation

3:   $A_{trans}\leftarrow [\begin{array}{ccc} 1 & 0 & x_{shift}\\ 0 & 1 & y_{shift} \end{array}]$

4:   $C_{aligned}\leftarrow \text{WarpAffine}(C,A_{trans})$    ▷ Apply coarse translation

5:   $ff\leftarrow \text{DenseOpticalFlow}(T,C_{aligned})$    ▷ Calculate optical flow

6:   $A_{refine}\leftarrow \text{EstimateAffineFromFlow}(ff)$    ▷ Refine from flow field

7:   $A_{final}\leftarrow \text{ComposeTransforms}(A_{refine},A_{trans})$    ▷ Combine coarse and fine transforms

8:   **return** *A*_*final*_

9: **end function**

#### 4.2.4 Super-resolution.

Next, a super-resolution technique is applied to produce a *super-template* from the set of matched candidates. The rationale is that by fusing information from all candidates, we can create a higher-quality template with sub-pixel detail that is more robust to noise and artifacts, thereby improving subsequent matching steps. The high-level functioning of the process is described in Algorithm 2.

**Algorithm 2 Super-template generation.**

**Require:** Set of candidate coordinates $P=\{p_{1},...,p_{N}\}$

**Require:** Set of affine transformations $A=\{a_{1},...,a_{N}\}$

**Require:** Full input image *I*

**Require:** Upscaling factor *s*

**Ensure:** Super-template *T*_*super*_

1: **function** GENERATESUPERTEMPLATE(*P*,*A*,*I*,*s*)

2:   C←ExtractCandidatePatches(I,P)    ▷ Extract candidate patches and   prepare grid

3:   G← Coordinate grid of shape (h·s,w·s)

4: $C_{upscaled}\leftarrow \emptyset$

5:   **for** *i* = 1 to *N* **do**

6:    $a_{i}^{-1}\leftarrow \text{InvertAffine}(a_{i})$

7:    $G_{aligned}\leftarrow a_{i}^{-1}(G)$    ▷ Project high-res grid to candidate local frame

8:    $G_{source}\leftarrow G_{aligned}+p_{i}$ ▷ Translate to local image coordinates

9:    $c_{i}^{\prime }\leftarrow \text{InterpolateIDW}(I,G_{source})$    ▷ Interpolate pixel values from full source image

10:    $C_{upscaled}\leftarrow C_{upscaled}\cup \{c_{i}^{\prime }\}$

11:   **end for**

12: $T_{super}\leftarrow \text{RobustAverage}(C_{upscaled})$    ▷ Aggregate color information and filter outliers

13:   **return** *T*_*super*_

14: **end function**

The process begins by defining a high-resolution grid of pixels, upscaled from the original candidate size by a given factor. For each candidate, we project this grid back into the source image’s coordinate system using the inverse of the affine transformation determined during the *registration* step. This projection yields a set of non-integer, sub-pixel coordinates. A color value for each of these coordinates is then estimated using an inverse distance weighting (IDW) interpolation scheme. This method calculates a weighted average of the four nearest neighboring pixels, where each weight is inversely proportional to the Euclidean distance from the sampling point to the pixel’s center. This procedure generates a distinct up-scaled image for every candidate, which is then averaged, excluding outlier pixels that deviate excessively from the mean, which are considered noise.

#### 4.2.5 Induction.

Our template matching pipeline repeats the previous steps several times to use as much information as possible to precisely detect all patterns. Still, template matching may fail to find patterns that are very different from the initial reference template. For instance, this is likely to happen due to a worn-out effect applied only on a few patterns or because patterns on the edge of the fabric are slightly distorted by the lens, slightly misplaced, or partially cut. Our specific use case benefits from the assumption that logos are placed on the fabric in a grid-like pattern (though not necessarily rectilinear), forming a lattice of points. To make our method more robust, we exploit this assumption in an *induction step*, outlined in Algorithm 3, which extrapolates the positions of missing patterns by relying on the geometry of the detected grid.

**Algorithm 3 Induction of missing patterns.**

**Require:** Set of detected pattern center coordinates (image plane) *D*

**Require:** Grid parameters (distances between logos and respective angles) *α*

**Require:** Search area boundaries *β*

**Ensure:** Set of induced pattern coordinates *I*

1: **function** INDUCEMISSINGPATTERNS(D,α,β)

2:   $C_{image},C_{canon}\leftarrow \text{FindReferencePairing}(D,\alpha )$ ▷ Find mapping (image and canonical planes)

3:   $T_{global}\leftarrow \text{EstimateAffine2D}(C_{canon},C_{image})$ ▷ Estimate a single global transform

4:   $G_{canon}\leftarrow \text{GenerateFullCanonicalGrid}(T_{global}^{-1},\beta )$ ▷ Generate the full theoretical grid

5:   $G_{canon}^{matched}\leftarrow \text{FindNearestInGrid}(C_{image},T_{global}^{-1},G_{canon})$

6:   $G_{canon}^{induced}\leftarrow G_{canon}⧵G_{canon}^{matched}$ ▷ Identify missing points

7:   I←∅

8:   **for** each point *p*_*canon*_ in $G_{canon}^{induced}$ **do**

9:    $N_{canon}\leftarrow \text{FindKNearestNeighbors}(p_{canon},G_{canon}^{matched})$

10:    **if** $|N_{canon}|\geq \text{min\_neighbors}$ **then** ▷ min_neighbors = 6 in our implementation

11:     $N_{image}\leftarrow \text{GetCorrespondingImagePoints}(N_{canon},C_{image},C_{canon})$

12:     $T_{local}\leftarrow \text{EstimateAffine2D}(N_{canon},N_{image})$

13:     $p_{induced}\leftarrow \text{ApplyTransform}(T_{local},p_{canon})$

14:     $I\leftarrow I\cup \{p_{induced}\}$

15:    **end if**

16:   **end for**

17:   **return** *I*

18: **end function**

The process begins by establishing a correspondence between the detected pattern centers in the image (the image plane) and an idealized, integer-coordinate grid (the canonical plane). Starting from a randomly selected candidate with a complete neighborhood (*i.e.*, whose four neighbors exist and have been found), it recursively searches for candidates around their expected position and maps all connected patterns to their corresponding canonical coordinates. From this set of point correspondences, a single global affine transformation is estimated to model the overall rotation, scaling, and shearing of the entire lattice, which may be caused by fabric folding or the image stitching process. We use the estimateAffine2D function from OpenCV to compute this transformation. It is then used to project a complete, theoretical grid from the canonical plane back onto the image, creating a theoretical “scaffold” of all expected pattern locations. The points on this scaffold that do not align with any previously detected patterns on the image plane represent the missing candidates that were extrapolated (or induced).

However, a single global transform cannot account for localized stretching or warping of the fabric. Therefore, a refinement is performed for each missing candidate by calculating a new, local affine transformation. This local transform is more precise because it is computed using only the nearest detected neighbors of that specific candidate. Applying this tailored, local transformation to the candidate’s canonical coordinates yields a highly accurate final position in the image, effectively inducing the missing patterns while respecting local geometric variations.

### 4.3 Operational phases

Having detailed the core components of our pipeline, we now describe their organization into our two operational phases. As introduced in Sect 4.1, our method consists of a *calibration* phase to learn optimal hyperparameters for each core component and an *inference* phase to quickly detect patterns. This two-stage design is advantageous because it separates the time-consuming optimization process from the rapid detection step. After an initial calibration, the optimal parameters are saved, allowing the inference to be run quickly and repeatedly. This is particularly useful in industrial settings where the same logo may appear on different materials with minor variations in color or texture.

#### 4.3.1 Template selection and calibration.

**Fig 6:**
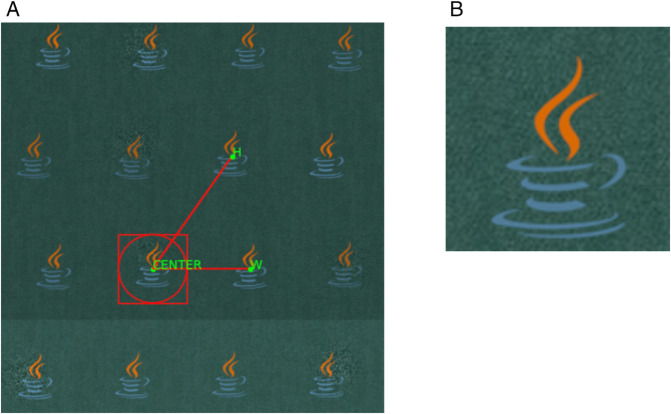
The manual template selection process and the resulting cropped template. (a) Template selection process via GUI. (b) The selected initial template.

**Fig 7:**
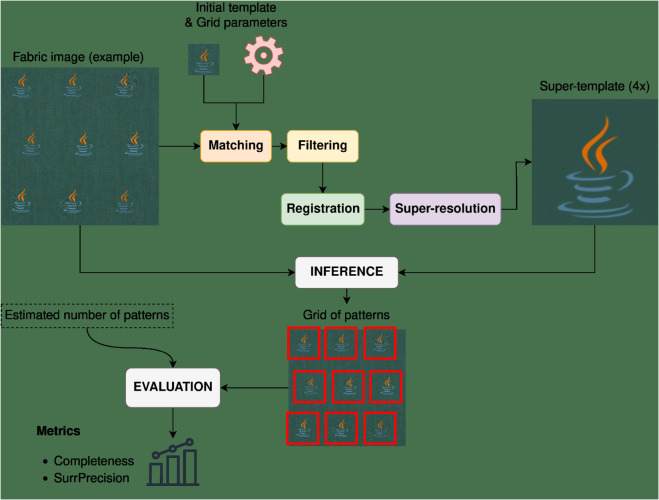
The calibration process, which is repeated for different configurations of hyperparameters.

The calibration phase (Fig 7) is the most computationally intensive step, designed to find the optimal configuration for a new material. It begins with a manual *template selection* via a GUI (Fig 6), where the user selects three adjacent logos. This defines the *initial template* and provides an estimate of the grid geometry (distances, logo radius, and angles) required for the induction step. The user also provides an estimate of the total number of patterns present in the image, *n*_*t*_.

The performance of the steps described in Sect 4.2 depends on several hyperparameters that must be carefully chosen. For example, the NCC threshold in the matching step controls the number of candidates, while various strategies and thresholds in the filtering phase can be used to discard candidates more aggressively. Similarly, the DIS optical flow method offers parameters that create a trade-off between speed and performance.

The calibration phase runs the complete pipeline with different parameter combinations and selects the configuration that yields the best results. The optimal algorithm should: (a) identify all patterns in the input image, and (b) pinpoint their centers with high precision (*i.e.*, ≤ 1 px error). To evaluate the first objective, we use the *completeness* score defined in Eq 2. This metric compares the number of detected patterns, *n*_*p*_, against the user-provided estimate, *n*_*t*_, and is designed to symmetrically penalize both over-detection and under-detection.

$$\text{Completeness}(n_{p},n_{t})=\frac{\min(n_{p},n_{t})}{\max(n_{p},n_{t})}$$ (2)

Relying solely on completeness during calibration would favor configurations that produce numerous, yet poorly aligned, matches. To counteract this bias, we introduce a *surrogate precision* metric. This is defined as the median NCC score between the final super-template and all detected candidates. Since ground-truth position data is unavailable in our case, this score serves as a proxy for alignment quality, where a higher score indicates better quality. This metric is defined in Eq 3, where $C=\{c_{1},c_{2},...,c_{n_{p}}\}$ is the set of matched candidates and *ncc*(*c*_*i*_,*T*) represents the NCC score between a candidate *c*_*i*_ and the super-template *T*. Since they have identical dimensions, this score is calculated as the zero-offset value *R*(0,0) from Eq 1, where *I* is replaced with *c*_*i*_.

$$\text{SurrPrecision}(C,T)=\text{median}(\{ncc(c_{i},T):1\leq i\leq \left|C\right|\})$$ (3)

Finally, we combine these two scores using their harmonic mean, much like the widely used F_1_-score. The calibration process concludes by selecting the hyperparameter configuration that maximizes this combined measure. The full process is outlined in Fig 7.

#### 4.3.2 Inference.

**Fig 8:**
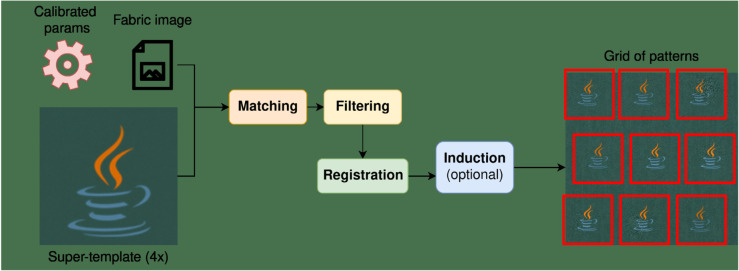
The streamlined inference process utilizes the calibrated configuration to detect fine candidates.

The calibration phase yields the optimal hyperparameters and the high-quality super-template for a specific “registered” fabric. In the subsequent inference phase (Fig 8), this saved configuration is used to swiftly process new images of the same material.

During inference, the pre-computed super-template is directly matched against the image, followed by filtering, registration, and an optional induction step. By eliminating the need for the iterative parameter search and the time-consuming super-resolution step, this approach significantly accelerates the final detection process.

### 4.4 Speed optimizations

To ensure our method is viable for our industrial application, we implemented several speed optimizations for both the calibration and inference phases. We leverage multiprocessing to parallelize computationally intensive tasks; for instance, running multiple calibration trials simultaneously. For the calibration phase, we also experiment with down-scaling the input image by different factors to reduce training times, as these are typically dependent on the image size (and the number of patterns). Furthermore, during inference, we divide the image into four partially overlapping tiles, one for each corner, and execute the inference concurrently on each tile, a process we call *tiling*. Beyond these architectural optimizations, we also investigate parameters that create a trade-off between speed and precision. These include the configuration of the optical flow method and the number of degrees of freedom when estimating the affine transformation from point sets (4 for a partial transform, 6 for a full affine transform). The performance implications of these optimizations are discussed in the following section.

## 5 Experiments

This section details the experimental evaluation of our proposed method. We compare its performance against several publicly available solutions on both a custom synthetic dataset and real-world images from our industrial setting. Lastly, we conduct a qualitative analysis using a diverse set of publicly available fabric-like images, a selection of which is presented in Appendix B, with additional examples available in the Supporting information B.

### 5.1 Synthetic dataset

A significant challenge in evaluating pattern detection algorithms is the lack of public datasets suitable for our use case. To the best of our knowledge, no existing dataset for repeating patterns on fabric provides ground-truth annotations for the pattern’s center coordinates (see Table 2). While we collected a small set of images from our industrial partner, these also lack annotations and cannot be publicly released. Unfortunately, manual annotation is impractical for this task, as human operators cannot reliably determine pattern centers with the sub-pixel accuracy required for a rigorous evaluation.

Therefore, to enable a precise and quantitative assessment of our method’s sub-pixel accuracy against competing baselines, we generated a synthetic dataset of five images. This approach gives us complete control over the ground-truth data, allowing for an unambiguous performance evaluation. The images were also augmented to simulate challenging real-world conditions, such as significant image noise and uneven lighting. To closely model scenarios from the textile industry, we construct the images by overlaying logos of varying complexity onto seamless backgrounds resembling leather and fabric textures. These logos were arranged in a near-regular grid. To increase the dataset’s complexity and better reflect real-world challenges, we apply several geometric and photometric augmentations that affect both the grid and the individual logos:

- **Alternating rows**: rows are slightly offset, creating a staggered effect (similar to a brick wall) rather than a square grid;
- **Random translation**: each logo has a chance to be randomly translated by a small amount around its center, further disrupting the repetition pattern;
- **Random rotation, scale and contrast**: logos may also be (very slightly) rotated, up- or down-scaled, and have their contrast slightly changed;
- **Gaussian and additive noise**: each logo has a small chance of having noise added to it, both in the form of additive (salt and pepper) noise as well as Gaussian blurring. These noise effects are distributed in a small normal distribution with its center randomly placed around the logo;
- **Bands of contrast**: horizontal bands of the image can have their contrast slightly changed to simulate uneven lighting and/or image stitching.

**Fig 9:**
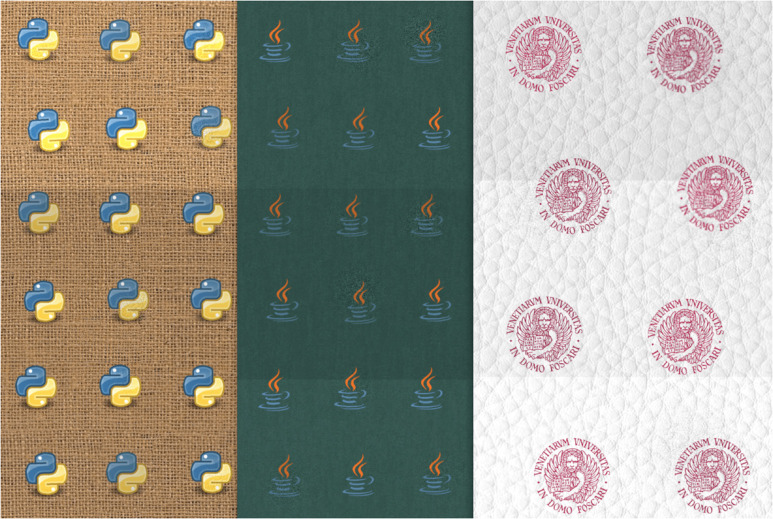
Cropped samples from our synthetic dataset. Each image consists of logos overlaid onto a fabric-like texture, with various types of synthetic noise and augmentations applied.

Such transformations produce challenging examples, which we deem sufficient to comprehensively evaluate our method in most situations that might arise in real-world scenarios. Fig 9 illustrates some of the patterns and textures used in our synthetic dataset.

We note that the real-world images from our industrial collaborator cannot be disclosed publicly due to the presence of trademarked designs. Moreover, the images lack the ground-truth annotations necessary for quantitative evaluation. The development of our synthetic dataset was therefore essential for ensuring the transparency and reproducibility of our findings. Although our synthetic dataset is small, its size reflects the operational reality of our industrial partner, who works with a limited number of brands and can provide only a sparse set of examples. This data-scarce environment is a core constraint of the problem, highlighting the unsuitability of conventional supervised methods that depend on large-scale training corpora.

The logos are created using open-source logos of well-known programming languages and our university logo. Specifically, the images will be named ‘golang’, ‘java’, ‘rust’, ‘python’, and ‘unive’ (University of Venice), reflecting the origins of their logo. We release our dataset and the code used to generate it in this repository: https://github.com/ebruayyurek1/synthetic-fabric.

### 5.2 Tested methods

As mentioned in Sect 1.1, our specific use case is designed to be applied to a concrete industrial setting with several constraints (speed, precision, and lack of training data). Notably, these constraints vastly restrict the methods that can be applied. Firstly, most recent proposals are based on machine learning models trained extensively on large amounts of data. However, this situation does not align with our scenario. Secondly, unsupervised FRPD methods are often not suitable as out-of-the-box solutions because (*a*) patterns are almost always extracted automatically, with a focus on their repeating nature and not on the precise center discovery, and (*b*) the fact that many of these methods struggle in the presence of the common nuances of textile or manufacturing materials. Though the latter point has been addressed by various methods (such as [143]), they are still difficult to apply to this specific scenario.

We identified three main classes of candidate methods:

- TM-based approaches;
- DL-based Object Detection;
- Near Regular Texture and Deformed Lattice Detection.

We now discuss these classes and describe, whenever applicable, methods we utilized from this class.

#### 5.2.1 Template matching.

As discussed in Sect 2.2, TM is one of the most basic techniques in CV for object detection. Though simple, TM can achieve good results, and we report it as a baseline approach. We also include a more expensive but also more precise variation that applies small random transformations to the template and matches the multiple results templates across the image (hereinafter referred to as TM++).

We also considered refined approaches using geometric feature matching. While methods like SIFT+RANSAC offer invariance to scale and rotation, applying them directly to the entire image is problematic. The uneven nature of fabric generates numerous spurious keypoints, and traditional matchers struggle with the one-to-many matching required for multiple logo instances. To address these issues, we investigated two strategies. The first, which attempted to group keypoints using density-based clustering (DBSCAN), was unsuccessful due to the high number of spurious features. The second strategy was a hybrid, two-step approach. First, a permissive TM step (in terms of NCC threshold) performs a coarse localization, identifying candidate regions where logos might be present. Second, for each candidate, a feature-based refinement step using SIFT+RANSAC is applied. This step precisely aligns the template to the candidate region by matching local features, correcting for the slight warping of logos and yielding a more accurate bounding box. We found this hybrid approach to be the most promising. However, its effectiveness was ultimately limited by the descriptor’s lack of robustness in some instances, and the computational expense of the feature extraction and matching process rendered it unsuitable for our target use case. To substantiate these findings, we include the results of this hybrid approach in Sect 5.4 to demonstrate its limitations.

#### 5.2.2 DL-based object detection.

In recent years, object detection methodologies in CV have been predominantly influenced by DL strategies, particularly those based on CNNs.

However, the application of DL-based methods to our specific use case can be challenging, first and foremost due to the variability in textures and logos. Additionally, these networks necessitate extensive training data in order to work correctly. Though this can be mitigated through transfer learning, some degree of fine-tuning remains necessary, complicating the application to entirely novel datasets.

As logos in industrial settings can change frequently, the most feasible approach in this context is to attempt to identify them as geometric shapes, thereby differentiating them from the background material. While this method may not fully satisfy the requirements of our use case, we have identified some methods that we deem fair to test. CNN-based methods represent the current state-of-the-art in this field, and some works reference foundational methods such as Mask-RCNN and YOLO [104,109,144,145]. The work by [104] proves interesting for our use case, as it builds on such networks. Although the objective of this work differs from ours (*i.e.*, detection of texels and extraction of individual texel attributes), the authors provide a fully annotated element-based texture dataset. We adapt this dataset to our use case, developing a YOLO-based method trained on the proposed dataset, and subsequently assessing its transfer learning capabilities.

#### 5.2.3 Cellpose.

Cellpose [146,147] is a versatile algorithm originally designed for cellular segmentation. The original version of Cellpose can accurately segment a diverse range of cell images without the need for model retraining or parameter adjustments.

Cellpose also includes generalized pre-trained models to enable zero-shot segmentation on a wide range of images and can optionally enhance noisy images to improve segmentation results [147]. While traditionally used in biomedical applications, Cellpose has found usage in various other domains [148]. In this study, we utilize Cellpose 3, leveraging the image restoration feature to augment the input images. We employ the generalist cyto-v3 model, which is recommended for general applications, in conjunction with the denoising functionality provided by the latest version. We provide an estimate of the diameter of the structures to be segmented using the radius of the *initial template* that was selected by the user. Following the segmentation and center computation, we select all pairs of instances whose distance is less than the radius and merge them into a single center by averaging. Cellpose tends to segment parts of logos as separate instances, making this post-processing step necessary to identify the most probable center by aggregating entities.

#### 5.2.4 Other methods tested.

To evaluate our pipeline, we attempted to implement several publicly available methods. Though we were not successful in their implementation, we believe it is valuable for future research to document the challenges we encountered. This section briefly discusses each of them.

- The authors of [108] propose a method that detects repeated patterns on a grid within a single image, leveraging the activations of pre-trained CNN filters at various layers. The published code did not work correctly on our images, due to the non-rectilinear grids.
- Similarly to [104], the method described in [99] also uses activations of a pre-trained CNN to detect repeated patterns. Unfortunately, we encountered several challenges with this approach that made it impossible for us to replicate it. Most notably, these include outdated code and difficulties in accessing the trained model weights.

### 5.3 Metrics

To measure the performance of pattern detection on our dataset, we match each true center to a predicted center. To this end, we look for predicted centers in a radius equal to 5% of the distance between adjacent centers. A match is only valid when exactly one predicted center can be found in such an area. Then, we adopt the following metrics to measure the performance.

**Root Mean Square Error (RMSE)**

quantifies the error (in pixels) between the predicted ($\hat{y}$) and true centers (*y*) and is defined in Eq. 4.

$$\text{RMSE}(y,\hat{y})=\sqrt{\frac{\sum_{i=0}^{N-1}(y_{i}-{\hat{y}}_{i}{)}^{2}}{N}}$$ (4)

**Precision**

is an additional metric generated using RMSE (Eq. 5), where *radius* is the search radius used during the matching phase. Here, the RMSE cannot be higher than the search radius, hence this metric is guaranteed to be in [0−1]. We use this measure in the *fitness* computation described below.

$$\text{Precision}(y,\hat{y})=1-\frac{\text{RMSE}(y,\hat{y})}{radius}$$ (5)

**Completeness (COMP)**

is defined in Eq 2 and quantifies the ratio of patterns that were detected, out of the total number.

**Fitness (FIT)**

combines *precision* and *completeness* with an harmonic mean. This global score represents our final metric, similar to an F_1_ score.

### 5.4 Results on synthetic data

Table 3 reports the metrics for all the tested methods. Times are noted with the mean and standard deviation measured over 5 runs, using the best calibration parameters.

**Table 3:** Test metrics on the synthetic dataset (average of 5 runs). Std. dev. is reported in brackets. ↓ means lower is better, while ↑ means higher is better.

Model	RMSE ↓	COMP ↑	FIT ↑	Runtime (s) ↓	Training time ↓
TM	0.634 [±0.13]	0.940 [±0.11]	0.942 [±0.06]	1.287 [±0.43]	N/A
TM++	0.555 [±0.12]	0.990 [±0.02]	0.972 [±0.01]	73.088 [±38.60]	N/A
TM-SIFT	1.670 [±0.11]	0.868 [±0.03]	0.750 [±0.02]	12.878 [±0.50]	N/A
TM-ORB	1.050 [±0.00]	0.828 [±0.00]	0.800 [±0.00]	9.910 [±0.557]	N/A
TM-BRISK	0.993 [±0.00]	0.924 [±0.00]	0.858 [±0.00]	12.159 [±0.627]	N/A
TM-FREAK	0.634 [±0.00]	0.974 [±0.00]	0.920 [±0.00]	10.707 [±0.25]	N/A
Cellpose (v3)	6.422 [±7.70]	0.827 [±0.25]	0.753 [±0.23]	2.258 [±2.26]	N/A
YOLO-ElBa	6.244 [±1.91]	0.778 [±0.27]	0.602 [±0.17]	1.218 [±0.36]	∼3 days
Our	0.321 [±0.13]	0.964 [±0.08]	0.967 [±0.04]	1.389 [±1.34]	2100 [±892] s

**Fig 10:**
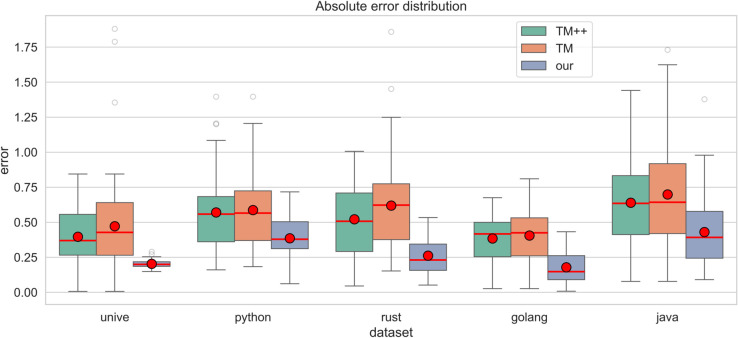
Comparison of RMSE (alignment) error over three tested methods (median = red line, mean = red circles).

We also compare the distribution of errors in terms of pixel alignment across our sample of test images. Box plots showing the distribution for the top three methods on each image are shown in Fig 10. For clarity of visualization, the results for YOLO and Cellpose, as well as for the descriptor-enhanced TM approaches, are not shown here, as errors are substantially higher, as also visible in Table 3.

A Mann-Whitney U test validates the hypothesis that our method has a significantly smaller error than TM++. We correct the *p*-values using the Benjamini–Hochberg procedure to mitigate the false discovery rate. Statistics are reported in Table 4 with *p*-values and effect sizes. We use the rank bi-serial correlation as effect size, with the formula by [149]. All measurements have *p*-values lower than 0.01.

**Table 4:** Test statistic for the hypothesis that our method has a lower precision error, based on measurements from Table 3.

Test	*p-value*	Effect size
unive	0.0	0.622
python	0.0	0.424
rust	0.0	0.609
golang	0.0	0.664
java	0.0	0.437

#### 5.4.1 Inference hyper-parameters and induction.

**Fig 11:**
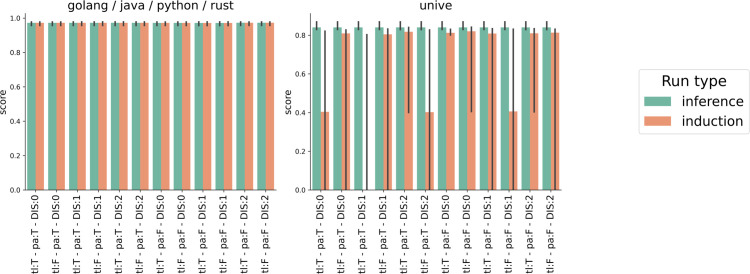
Fitness scores using various inference hyperparameters.

Fig 11 presents bar plots of the fitness scores for the test images, obtained using all possible combinations of hyperparameters during inference.

The figure is structured as follows. On the *x* axis, tl (tiling) and pa (partial affine) can be either T(rue) or F(alse), while DIS refers to the configuration of the DIS method (0 is the fastest, 2 is the slowest but most precise).

Experiments are run three times for each configuration with different down-scaling factors of the input image. Bars represent the median values over all runs for each configuration, with error bars marking the 95th percentile. The results for the ‘golang’, ‘java’, ‘rust’, and ‘python’ synthetic images are practically identical, so we only report results for the latter to simplify Fig 11.

**Fig 12:**
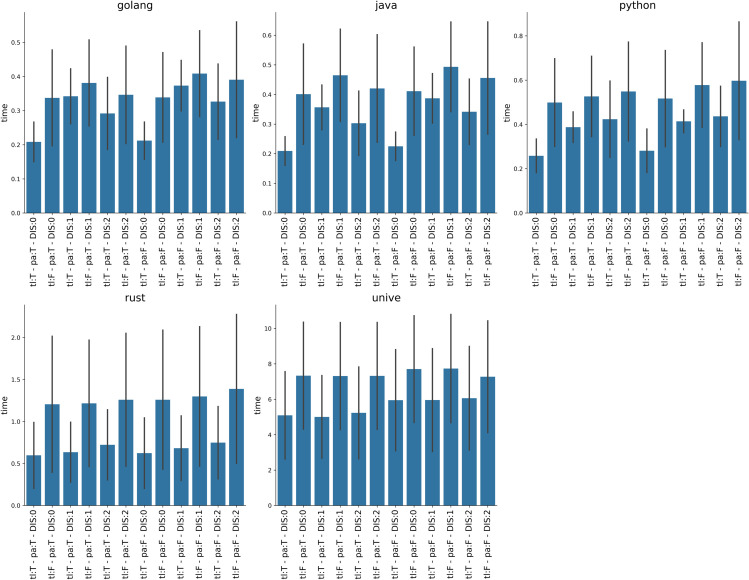
Inference time in seconds across different configurations. These are defined by the use of tiling (tl), partial affine estimation (pa), and various settings for the DIS optical flow method. Error bars indicate the 95th percentile of the measured times.

We observed that, in most cases, the hyperparameters do not significantly affect the inference phase, which validates the robustness of this approach. The induction process, however, is less stable, with results varying significantly between different runs. This variability in the ‘unive’ image is attributed to its low contrast, which makes pattern detection more challenging. Considering the overall results, we selected the configuration tiling: True, partial affine: False, DIS: 0 as the default, as it demonstrated higher stability across all images. This also indicates that tiling, in addition to enabling parallelism during inference, does not degrade performance, and the fastest DIS configuration is sufficient to produce good results. The average inference time for each configuration (over 5 runs) is reported in Fig 12, with the standard deviation in the error bars.

#### 5.4.2 Induction step.

Fig 11 shows that *induction* did not provide any advantage on 4 out of 5 images, as the algorithm was already identifying all patterns correctly. The induction step improved COMP on the ‘unive’ image by finding all the missing patterns, but it significantly increased the RMSE error, as the estimated matches were not very precise. This is expected, as the induction step is designed to provide an estimate of the pattern position. This also explains why the fitness bar plots show lower values for the induction step on the ‘unive’ image.

**Fig 13:**
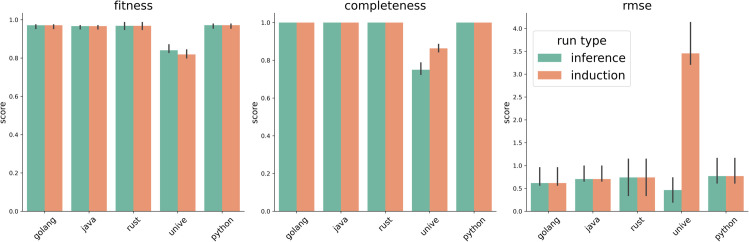
Effect of the induction step (in orange) during inference. Median scores over 5 runs are reported, with 95th percentile error bars, using the default inference configuration.

We also show the effect of this step over all three metrics by aggregating 5 runs in Fig 13. In these experiments, the inference hyperparameters were set to the default values explained in the previous section. The results show that, when considering the median scores, the induction step can improve COMP (by approximately 11% on the ‘unive’ image), but, as mentioned, the matches are not likely to be precise, leading to a substantially higher RMSE. For this reason, we did not use the results of the induction step for the rest of the experiments, limiting our comparisons to inference results.

Despite its limited utility on our synthetic images, we observed that *induction* remains beneficial in practical settings for estimating pattern positions, particularly on noisy images and at image boundaries where TM is less effective. This effect is evident in the supporting tests conducted on real data (see Sect 5.5), as well as the qualitative results presented in Appendix B and the Supporting information B.

#### 5.4.3 Super-resolution.

**Fig 14:**
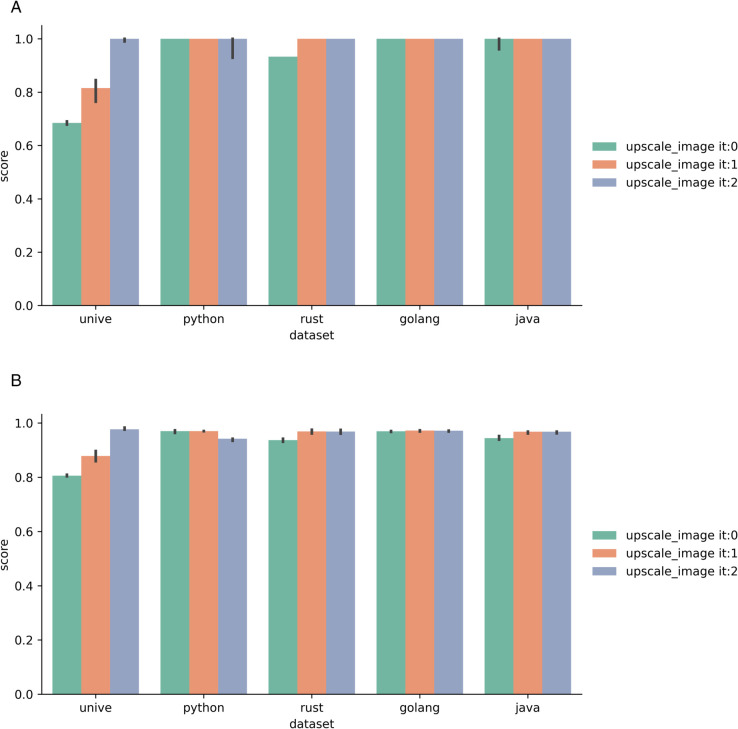
Comparison of completeness and fitness using 0, 1, or 2 iterations of super-resolution followed by matching-filtering-registration. Median scores are reported over 5 runs. (a) Completeness (COMP) values with different iterations. (b) Fitness (FIT) values with different iterations.

The core of our approach is the *super-resolution* step that provides an improved template to be matched. Furthermore, the *super-resolution, matching, filtering, registration* steps can be repeated several times to produce better candidates. Fig 14a and 14b show the FIT metric when calibrating using 0, 1, or 2 iterations. Results show the median score of 10 different calibrations with 5 different down-scaling factors, with error bars representing the 95th percentiles. Iterating the process helps in most cases, and more iterations lead to better COMP, although the usage of 1 or 2 iterations should be chosen empirically when the precision of the alignment is an issue. Note that using 0 iterations roughly corresponds to using TM followed by a single filtering and registration steps to produce the final candidates.

#### 5.4.4 Error analysis.

We perform an additional error analysis to investigate the robustness of our proposed method under several challenging image transformations that simulate adverse, non-ideal conditions. To this end, we synthetically degrade the test images from our synthetic dataset and evaluate the resulting increase in localization error (RMSE) compared to the baseline performance on the original images. As described in Sect 5.1, the synthetic dataset already incorporates a degree of artificial noise; this analysis, therefore, further tests the limits of our method under conditions that we expect to degrade performance significantly. Each image in the dataset is altered using one of the following transformations:

- **Uneven Illumination** (UI): Simulates shadows or vignetting by applying a linear intensity gradient across the image, modulating pixel brightness from 70% on one edge to 150% on the opposite;
- **Perspective Warp** (PW): Mimics the non-rigid nature of fabric by applying a subtle perspective warp, programmatically shifting the image corners inward by 2% of its width;
- **Rotation & Scale** (RS): Simulates minor camera misalignment and distance variations by applying a combined 2^°^ rotation and a 3% zoom-in;
- **Gaussian Blur** (GB): Simulates a loss of focus by convolving the image with a 5x5 Gaussian kernel to soften high-frequency details;
- **Gaussian Noise** (GN): Tests robustness against sensor imperfections by adding zero-mean Gaussian noise (standard deviation of 20) to each pixel channel, resulting in a substantially grainy image.

**Fig 15:**
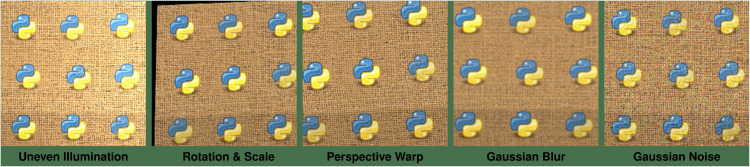
The effect of the various transformations, visualized on a cropped area from the python synthetic image. The type of augmentation is indicated below each image.

**Fig 16:**
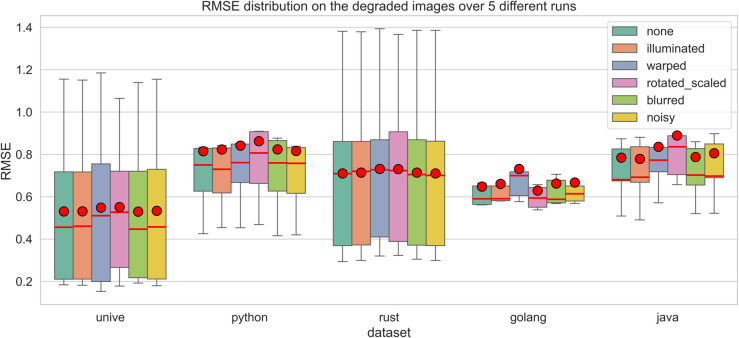
Boxplot showing the RMSE distribution over five different calibration configurations (each corresponding to a different input image rescale-factor), grouped by degradation type, with each configuration repeated five times. None represents the original image with no degradation.

Fig 15 shows the effect of these transformations on a sample image from our synthetic dataset. Table 5 summarizes the quantitative impact of each transformation on the localization error (RMSE). To ensure a robust evaluation, the results are averaged over five runs and across multiple configurations derived from different image rescaling factors. Specifically, this analysis includes not only the optimal configuration identified in our previous experiments but also four sub-optimal configurations that result from different, usually more aggressive, down-scaling of the input image before calibration. This is done to simulate a real-world scenario where an operator might prioritize a faster calibration time over maximum precision. The values in the table represent the percentage increase in RMSE relative to the baseline performance on the original, unaltered images. The overall distribution of RMSE scores across all these test conditions is shown in the boxplot in Fig 16.

**Table 5:** Increase in RMSE (%) on transformed images compared to the baseline. Lower values indicate higher robustness.

Dataset	UI	PW	RS	GB	GN
unive	0.01	3.34	3.84	0.23	0.07
python	1.03	3.28	5.78	1.08	0.37
rust	0.55	3.01	2.86	0.61	0.24
golang	2.00	12.83	2.90	2.18	1.48
java	0.70	6.54	13.40	0.41	2.85
**Average**	0.86	5.80	5.76	0.90	1.00

The results indicate that while our method is robust, its precision can degrade significantly in uncontrolled environments. On average, Uneven Illumination (UI), Gaussian Noise (GN), and Gaussian Blur (GB) introduce the smallest error increases, all at approximately 1% or less. This resilience is consistent with the properties of the NCC metric, whose normalized nature provides robustness against global changes in brightness and contrast. While the metric effectively handles global and small local variations, strong localized changes can still be detrimental. Conversely, geometric transformations that alter the spatial arrangement of the pattern prove more challenging. Perspective Warp (PW) and Rotation & Scale (RS) create the most significant misalignment, increasing the average RMSE to 5.80% and 5.76%, respectively. This behavior is characteristic of TM algorithms, which rely on a high degree of structural similarity that is directly disrupted by these geometric distortions. We note specific cases, such as the java example under rotation/scaling and the golang example under warping, where these effects are particularly pronounced and significantly impact localization precision.

Despite this increase, the RMSE remains within acceptable margins, generally corresponding to an offset of one pixel or less. While these small alterations were manageable, our analysis confirms that our approach is highly sensitive to significant changes in scale and rotation. Performance degrades rapidly under such conditions, as TM is not invariant to these geometric transformations. Moreover, Fig 16 shows that in a few instances, the localization error can exceed the 1-pixel threshold. These higher-error cases correspond to tests run with suboptimal configurations, specifically those using a more aggressive image downscaling factor to speed up calibration. We omit reporting the completeness (COMP) metric as some augmentations inherently shifted some patterns partially or entirely beyond the image boundaries, artificially lowering the COMP/FIT scores. This analysis highlights the significance of a controlled environment in our target industrial application, which mitigates these effects by ensuring uniform lighting and a flat, consistently aligned presentation of the material.

### 5.5 Results on real data

In addition to our experiments on synthetic images, we conduct tests using a collection of 20 real images of fabric materials featuring logos from well-known fashion brands, mostly sourced from our database. These images represent the fabric types relevant to our study, although we are unable to disclose them due to confidentiality constraints. Given the absence of annotated datasets for this specific task, these real images serve to further validate the robustness of our proposed method.

We selected images with a minimum resolution of 1200 x 1200 pixels and cropped them to achieve a roughly square aspect ratio. Each image was manually annotated to mark the coordinates of the centers of at least 20 logos. The annotation strategy involved selecting three logos from each corner, five logos around the center, and at least one logo from each side. This “sparse” annotation approach strikes a balance between the time-consuming dataset annotation and the utility of the benchmark. Notably, corners and edges present significant challenges for logo detection, as fabric patterns can exhibit slight variations that complicate recognition. To enhance our dataset, we generated an altered version of each image by adding random RGB noise, resulting in an additional 20 annotated images. An example of an annotated image is presented in Fig 18. We then evaluated our method against TM++, the best-performing baseline identified in our previous experiments, using the complete dataset. The average FIT, RMSE, and COMP metrics for both methods are summarized in Table 6.

**Fig 17:**
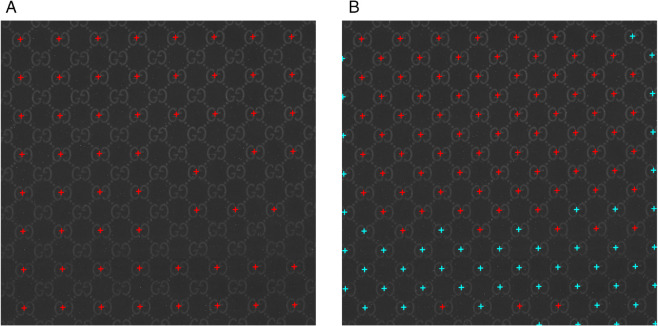
Comparison of pattern detection results on a real fabric image. (a) TM++ detection. (b) Detection with our method.

**Table 6:** Metrics on the manually annotated dataset of real images (average). Std. dev. is reported in brackets.

Model	RMSE ↓	COMP ↑	FIT ↑	Runtime (s) ↓	Training time (s) ↓
TM++	3.069 [±1.03]	0.607 [±0.28]	0.418 [±0.12]	53.891 [±26.25]	N/A
Our	3.628 [±0.99]	0.995 [±0.02]	0.567 [±0.09]	0.216 [±0.11]	8.025 [±1.51]

Image outputs of the detection step for one image are shown in Appendix A, Fig 17. More results are reported in the Supporting information B. Our findings clearly illustrate the advantages of incorporating the super-template step before matching, as it significantly increases the number of successful matches. Furthermore, the final induction step proves particularly beneficial for detecting logos that are partially visible at the edges of the images.

### 5.6 Discussion

#### 5.6.1 Synthetic dataset.

We begin our discussion with the results on the synthetic dataset. Our analysis is structured to address performance metrics and the selection of inference parameters for our proposed methods.

The comparison focuses primarily on TM++, the second-best performing method. Although the baseline TM also provides strong results within a reasonable time (see Table 3), our method outperforms it across all metrics. Regarding Cellpose and YOLO, we conclude that while they perform adequately in finding the approximate position of patterns, they cannot guarantee precise alignment without additional steps. YOLO’s requirement for a training dataset is a considerable disadvantage in our use case. For both neural methods, implementing further refinement steps is difficult to justify, as their performance is already inferior to the basic TM. Finally, feature-descriptor augmented TM approaches can provide a minor boost in COMP, but at the cost of a much longer inference time and worse overall RMSE and FIT scores.

**RMSE**

Our proposed methods demonstrate a significant improvement in terms of RMSE. The center alignment error is at least 43% lower (in RMSE) compared to TM++, the second-best method. On average, our method shows a 72% improvement over TM++, with an impressive peak of 118% improvement on one of the test images. The averaged metrics are summarized in Table 3. This improvement is also evident in Fig 10, which illustrates the distribution of error values (pixel offsets) using box plots. Our method consistently yields a lower error, a fact that is further substantiated by our statistical analysis presented in Table 4. In all cases, our method significantly outperforms the comparison method, TM++.

Our neural baselines (Cellpose and YOLO) are not competitive, primarily due to the limited training dataset, which substantially degrades their performance. This is evidenced by RMSE values nearly ten times higher than those achieved by a basic TM approach. Similarly, the descriptor-based augmentations are uncompetitive, as their efficacy is severely limited by the particular characteristics of fabric-based images. The best-performing method, TM-FREAK, achieves results comparable to a simple template matching algorithm that employs a less permissive NCC threshold.

**COMP and FIT.**

When considering COMP, TM++ is the best-performing method. However, our method competes closely, achieving an equal (sometimes better) score on 4 out of 5 images. The fitness score highlights a similar trend, being slightly higher on average for TM++. Despite this, TM++ falls short in terms of efficiency, taking a substantial 73 seconds on average compared to less than 2 seconds of inference time for our method, making TM++ practically unusable for our use case. We remark that TM++, like our method, also achieves a perfect COMP score on 4 out of 5 images, but it scores higher on the ‘unive’ example, which proves to be the most challenging for our method. It is worth noting that TM++ employs image augmentations (*e.g.*, small rotations, shifts, etc.) to enhance the detection performance. While this strategy proves effective when compared to standard TM, we intentionally avoided it in our method to minimize inference time. However, we acknowledge that incorporating some transformations could potentially improve our results, albeit at the cost of increased inference time. Indeed, our robustness tests in Sect 5.4.4 confirmed that changes in rotation and scale can degrade the precision of our method. Exploring these extensions is a potential area for future work.

Similar conclusions can be drawn for the other methods with respect to the COMP and FIT metrics. The neural methods perform considerably worse, while the descriptor-based augmentations generally underperform compared to the baseline TM approach. Notably, TM-FREAK achieves a better COMP score than the baseline, an improvement attributable to its lower NCC selection threshold. As previously mentioned, this is possible due to the keypoint validation step, which allows for a more coarse-grained template matching process. However, the FIT score for TM-FREAK is still lower than that of the basic approach.

**Runtime**

In terms of runtime, our method is comparable to YOLO and TM, with only a 14% increase on average, and never exceeding 6 seconds when using the default inference parameters. This means that even at its slowest, our method is still 6 times faster than the fastest runtime for TM++, which, as previously mentioned, is too slow for our use case. In the experiments, our method calibrates for an average of 35 minutes, with variations ranging from a minimum of 25 to a maximum of 61 minutes. All times are measured on an Intel Xeon Gold 5218R, with 20 parallel processes. In contrast, TM, TM++, and Cellpose do not require any training or extensive tuning, but they lag in terms of metric scores or inference time (in particular TM++). A similar limitation applies to descriptor-based augmentations, which rely on computationally expensive feature descriptor extraction, resulting in a substantially longer inference time.

#### 5.6.2 Inference parameters for our approach.

In Sect 5.4.1 we discussed a reasonable choice for default inference parameters, although, as mentioned, their selection is not critical for good results. The default parameters should provide an optimal balance between runtime and performance. As depicted in Fig 12, the inference time ranges from 0.2 to 11 seconds, depending on the image and the hyper-parameters. The ‘unive’ and ‘rust’ examples both use the approach that matches the super-template with the up-scaled image, using 2 and 1 super-resolution iterations respectively. The other three examples instead downscale the super-template to match the image resolution before the matching step. In practice, this means that the second matching step in the ‘unive’ example must handle four times the number of pixels compared to the first three examples. In the other three cases, even with 2 super-resolution iterations, the runtime does not exceed 1 second even in the worst-case scenario.

The choice of up-scaling or down-scaling, as well as the number of super-resolution iterations, significantly impacts the runtime. These choices are delegated to the calibration procedure to ensure optimal results. Indeed, we have observed that the up-scaling strategy might amplify defects or noise that is discarded when using a down-scaled super-template. Therefore, the best strategy should be determined on a case-by-case basis. In most scenarios, a single iteration should yield satisfactory results, especially if calibration time is a concern. Furthermore, Fig 12 shows that the use of tiling and DIS set with the fastest configuration (0) substantially reduces the average inference time.

The induction step, which operates in fractions of a second, can be utilized to estimate the position of missing patterns in instances of challenging examples with geometrical pattern displacement. The average runtime for this step is 0.04 seconds, peaking at 0.053 seconds. However, while this step can improve the detection rate (COMP), it cannot guarantee perfect alignment. We argue that, for our use case, it still offers a valuable tool to quickly infer pattern position, even when conducting a quick (albeit rough) calibration, particularly considering the extremely fast runtime.

Lastly, Fig 14a and 14b demonstrate the impact of the number of iterations on the COMP and FIT scores, respectively, when using the up-scaling strategy before matching. All the results listed previously were obtained with either 1 or 2 iterations, depending on which yielded the best tuning score during calibration. The bar plots reveal that increasing the number of iterations generally benefits FIT and COMP (+5% on average), with most images achieving perfect FIT scores using just a single iteration. Here, the average error also decreases with more iterations in all but one case. These findings validate our claim that our super-template-based approach enhances the quality of the matching step. While a single iteration suffices in most cases, two iterations could further improve results, though the optimal number should be ultimately determined by the calibration procedure, as previously argued.

#### 5.6.3 Real fabric images.

Our experiments on the real fabric image dataset demonstrate the strong performance of our method in terms of COMP (*i.e.*, recall) for logo detection (see Table 6). Specifically, our method achieves near-perfect detection rates for all logos, even under noisy conditions. The induction process plays a crucial role in this success, as it enables the identification of partial matches along the image edges, as illustrated in Appendix A. In terms of RMSE, both our method and TM++ yield similar results, with values falling within one standard deviation of each other. The slightly lower error observed for TM++ is likely a consequence of its lower detection rate; its COMP score is 38% worse than our method’s, indicating that it fails to identify more challenging patterns which could increase the average error. Overall, our method achieves a 35% higher FIT score while operating two orders of magnitude faster during inference. This highlights the practical advantages of our approach over a pure TM-based method, whose runtime makes it impractical for our use case.

### 5.7 Limitations

While our proposed method demonstrates robust performance, we acknowledge certain limitations. A primary limitation is the lack of inherent invariance to significant geometric transformations such as scaling, warping, and rotation. Although the method is effective against minor variations present in the images, its performance may degrade when faced with patterns exhibiting substantial geometric distortions. Future work could address this by incorporating techniques for rotation and scale-invariant feature extraction. Secondly, this work focuses on a specific class of fabric patterns characteristic of our industrial use case. Consequently, the findings may not be directly generalizable to all types of textiles or pattern-matching problems. The constraints and requirements of our target application guided the methodological design, and future research could evaluate the method’s performance on a broader range of fabric images with different pattern characteristics. Finally, our evaluation of neural network-based approaches was limited by the absence of a large-scale, annotated dataset. A valuable and significant direction for future work would be to undertake a dedicated data collection and annotation process. Creating such a public benchmark, while beyond the scope of the current study, would be crucial for the community. It would enable a definitive evaluation of deep learning models and could potentially unlock new levels of performance, establishing their viability for this industrial application.

## 6 Conclusion

This work presents a comprehensive review of the literature on fabric pattern matching, outlining the primary research tasks and organizing them within a proposed taxonomy. Our analysis reveals a notable scarcity of public datasets and reproducible benchmarks in this field. We acknowledge that this domain is of significant industrial interest, which may lead to restrictions on publishing proprietary experimental materials — a constraint also faced in this work. To help address this gap and foster reproducible research, we are releasing the synthetic dataset developed for this study, along with the generation code, which details the transformations applied to simulate real-world scenarios.

In addition to the literature review, we have presented a novel method for textile pattern recognition and benchmarked it against several existing methods, including TM and its variants, as well as transfer learning approaches using YOLO and Cellpose. Overall, our method achieves an effective balance between speed, precision, and semi-supervision, making it a robust and efficient solution for FPRD in an industrial setting. The method was validated on both our synthetic dataset and a manually curated dataset of real images. Our approach can detect repeated patterns with an average error of less than 0.5 pixels in just a few seconds, reducing the error by nearly half compared to other methods with comparable inference speeds. Moreover, it is designed to operate under strict industrial constraints, including high speed, sub-pixel precision, and a lack of extensive training data, confirming its viability for real-world deployment.

For future work, we could explore performing calibration on image crops to further reduce model setup time, a direction of practical interest that we have observed but not formally evaluated. We hope that this work will inspire further research and development in this field.

## A Qualitative results: Fabric images

**Fig 18:**
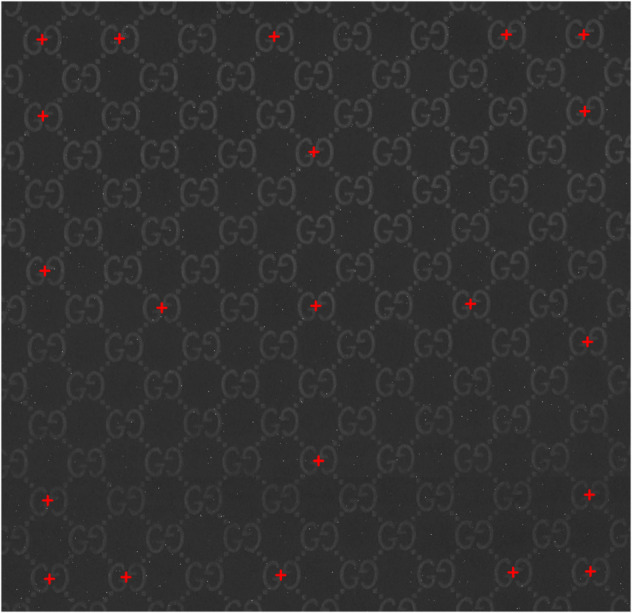
Example of a manually annotated real fabric image.

In Fig 17, we present the results of the FRPD task on the real fabric image dataset utilized for the benchmark described in Sect 5.5. This dataset was manually annotated by users experienced in the fabric-cutting process, which our work aims to support. We employed GIMP software https://www.gimp.org to pinpoint the coordinates of each logo as accurately as possible, acknowledging that the manual nature of this process may introduce some inaccuracies. An example of an annotated image is displayed in Fig 18. Additional examples of annotated images and further results can be found in the Supporting information B.

## B Qualitative results: Regular patterns

**Fig 19:**
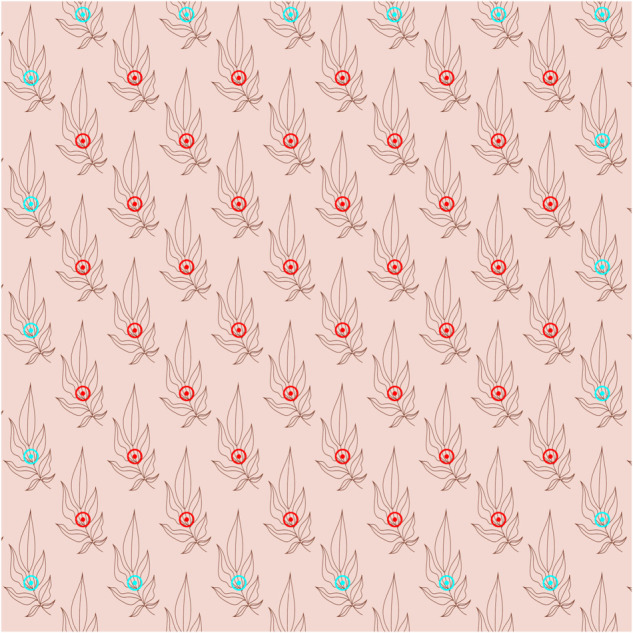
Detection of a floral pattern.

**Fig 20:**
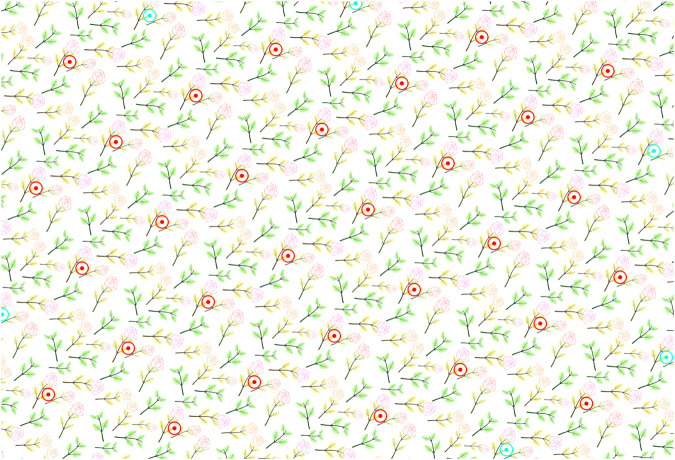
Detection of a complex floral pattern with several repeated elements (high-contrast for visibility).

We report the results obtained on two royalty-free images of patterns retrieved on Pixabay https://pixabay.com/images/search/fabric%20textures. More examples in higher resolution are reported in the Supporting Information B. The purpose of Figs 19 and 20 is to demonstrate the result of our FRPD method on realistic images. These examples are qualitative, as we do not possess any ground truth data. In Figs 19 and 20, centers detected using the inference process are represented by red circles, while the ones estimated by the induction process are denoted by light blue circles. These figures illustrate the impact of the *induction* step, which is particularly evident at the image borders, where it predicts patterns that are only partially visible. This underscores the significance of this step, even though such a scenario was not covered in our experiments on the synthetic dataset.

## Supporting information

S1 File Fabrics. Additional results from the application of our methodology to various patterns extracted from real fabric images of popular fashion brands.
(PDF)

S2 File Patterns. Further results of the application of our methodology to a variety of patterns.
(PDF)
