# Supplementary material for: Repeated pattern detection on fabric: A survey and novel approach
Source: PLoS One. 2026 Feb 5;21(2):e0340797. doi: 10.1371/journal.pone.0340797 (PMC12875591; doi:10.1371/journal.pone.0340797)

Repeated Pattern Detection on Fabric: a Survey and Novel Approach

Supplementary Material (real fabric images)

In this supplementary document, we present additional results from the application of our methodology to various patterns extracted from real fabric images of popular fashion brands.

Each example includes:

- the **annotated image** (ground truth) on the left;
- detected patterns using the **TM++ algorithm** in the center;
- detected patterns using **our algorithm** on the right, with centers estimated during the *induction* step highlighted in blue.

Centers are represented by cross markers, as outlined below.

Inference patterns

Induction patterns

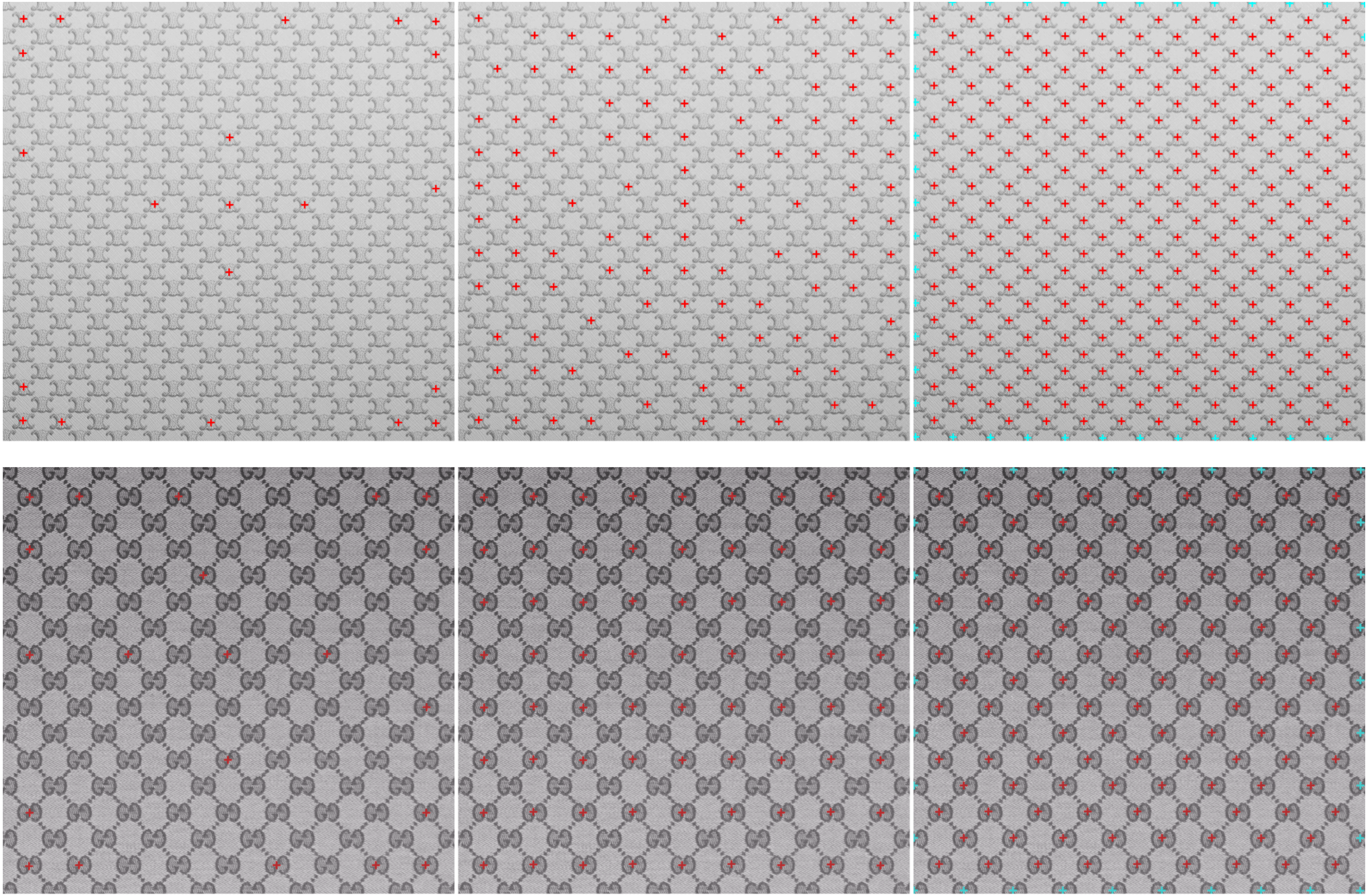

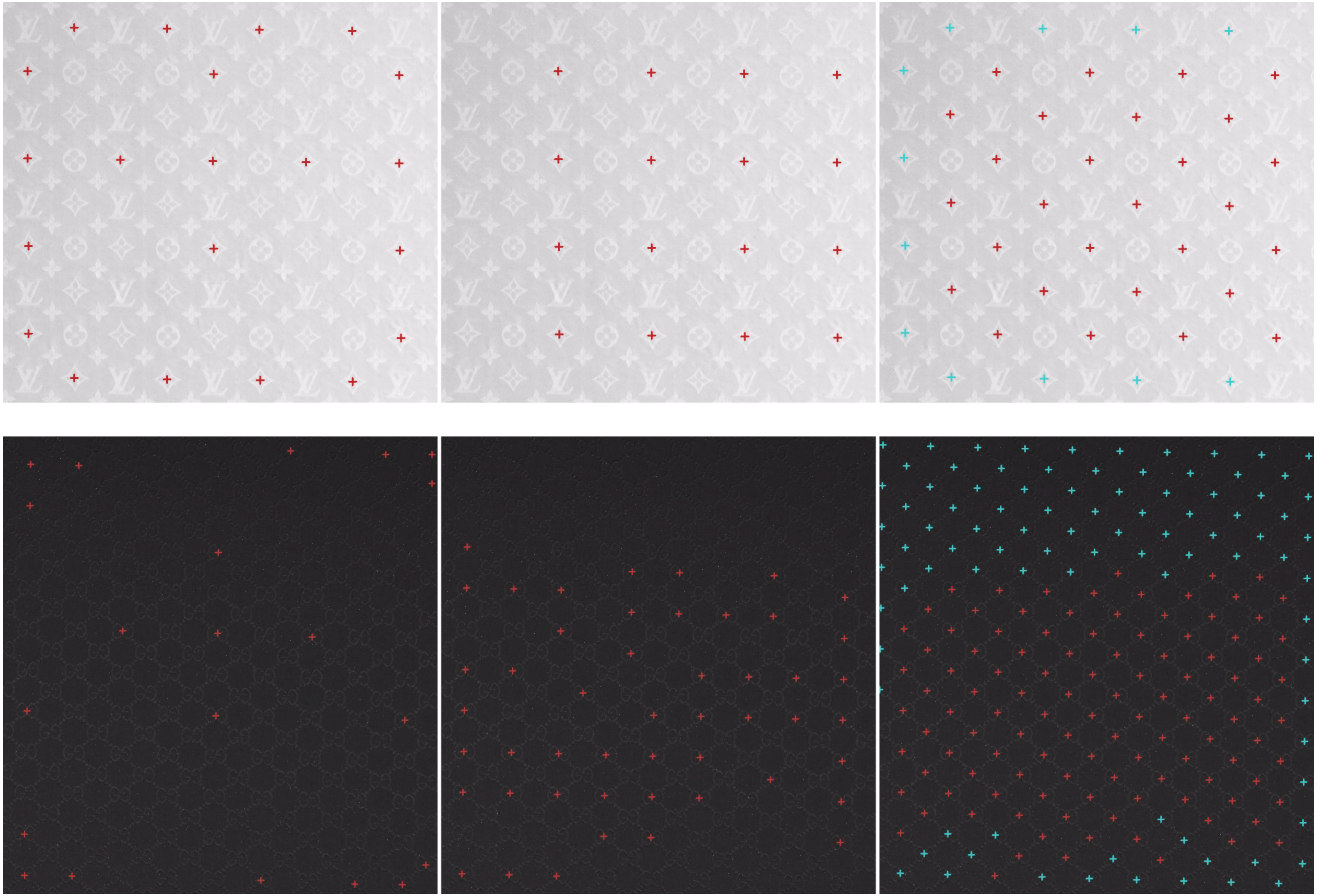

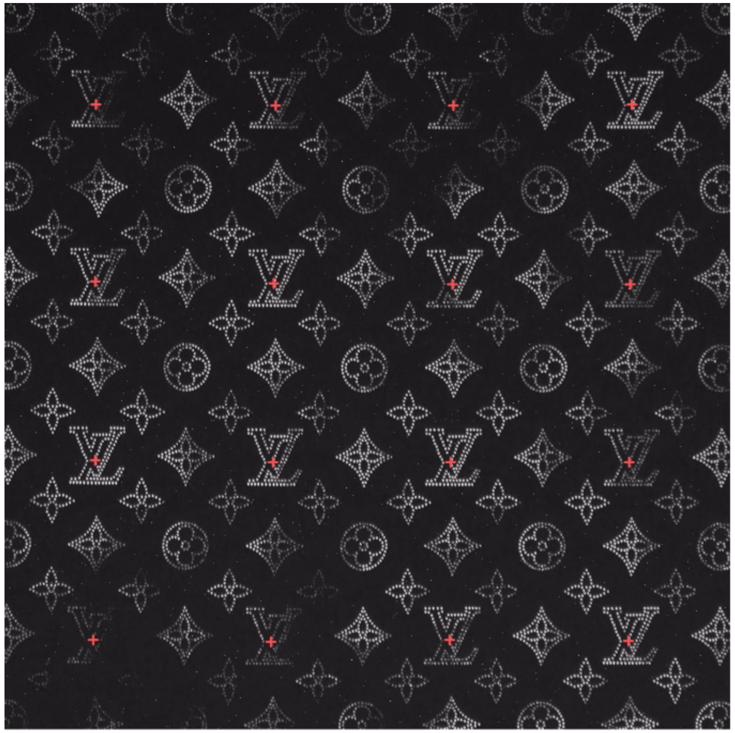
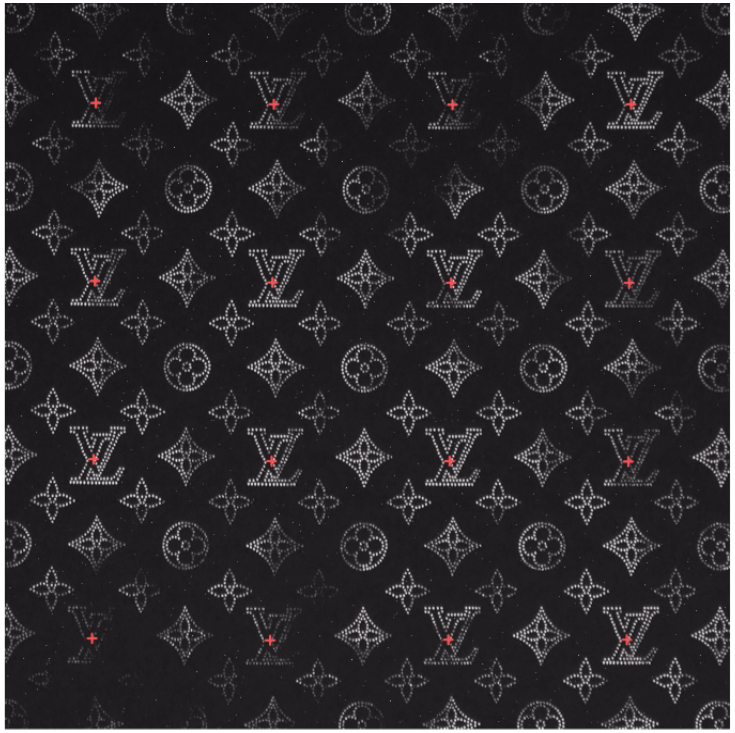
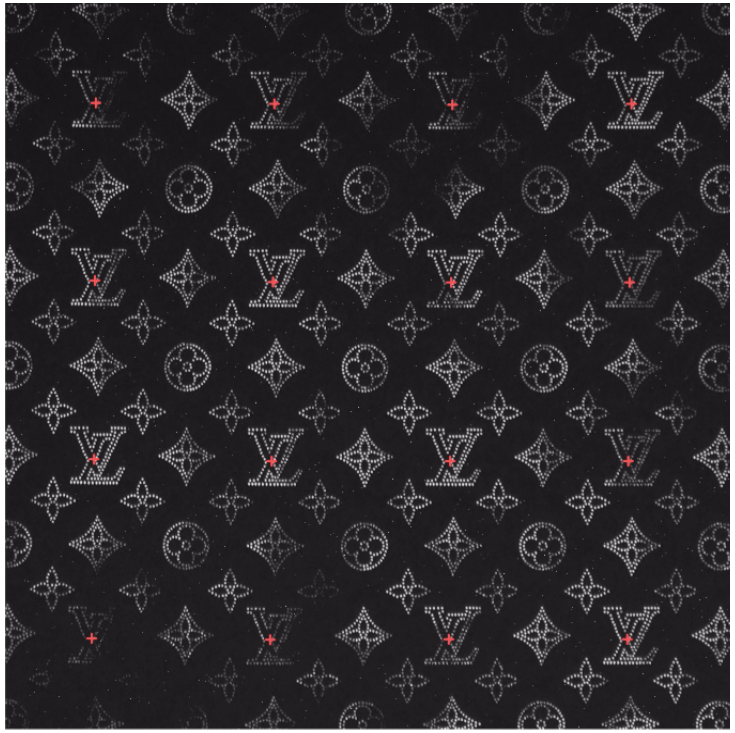
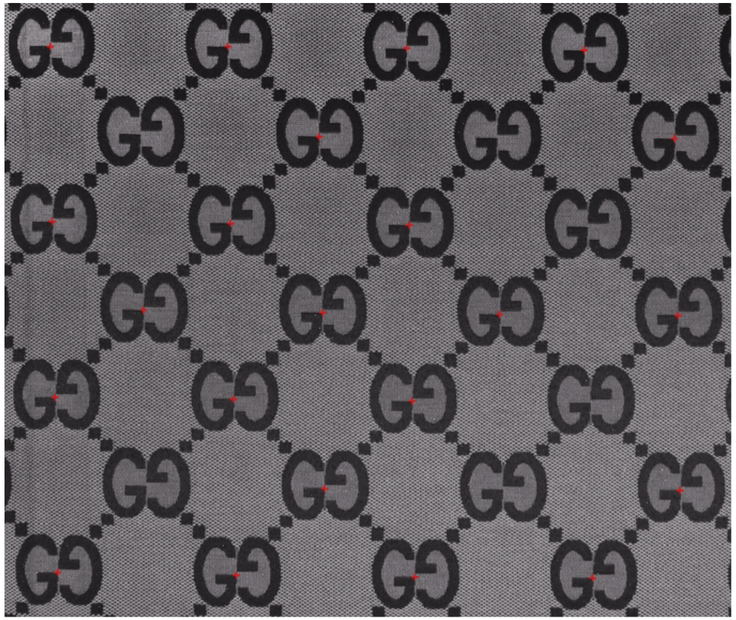
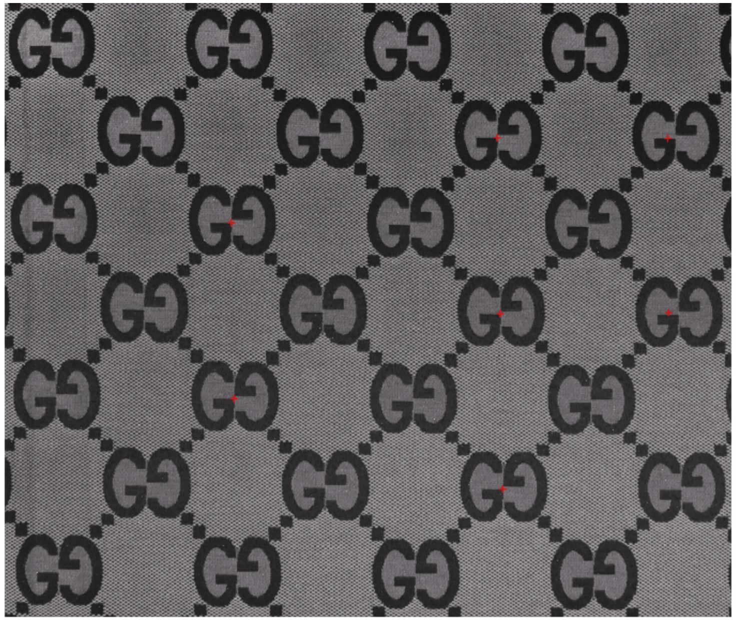
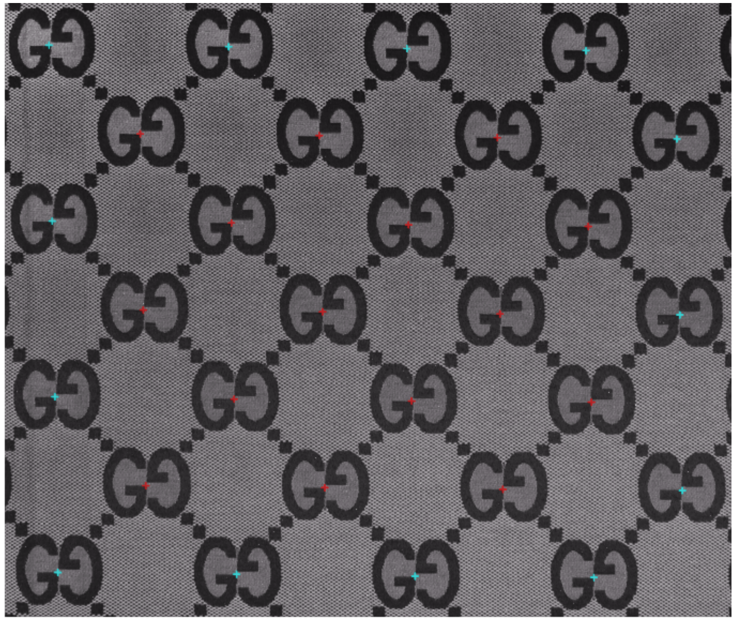

Supplement: S1 File — Additional results from the application of our methodology to various patterns extracted from real fabric images of popular fashion brands. (PDF) [file pone.0340797.s001.pdf]
