# Supplementary material for: Repeated pattern detection on fabric: A survey and novel approach
Source: PLoS One. 2026 Feb 5;21(2):e0340797. doi: 10.1371/journal.pone.0340797 (PMC12875591; doi:10.1371/journal.pone.0340797)
Supplement: S2 File — Further results of the application of our methodology to a variety of patterns. (PDF) [file pone.0340797.s002.pdf]

Repeated Pattern Detection on Fabric: a Survey and Novel Approach

Supplementary Material (pattern images)

In this supplementary document, we present further results of the application of our methodology to a variety of patterns. These examples utilize synthetic images sourced from Pixabay[1], given that the images from our primary application are proprietary and cannot be released publicly. All these images are royalty-free and licensed under the Pixabay Content License which allows free usage[2].
As we do not possess the coordinates of the centers for each pattern, these images serve as qualitative illustrations of the results. The quality of results can only be evaluated visually on actual images.

Each example displays the original image on the left and the identified patterns (centers) on the right. Remarkably, all results were obtained in under ten seconds of runtime, including inference and induction. The detected centers are represented by red circles (inference process), while the centers computed through the induction process are denoted by light blue circles.

Inference patterns

Induction patterns

[1] https://pixabay.com/images/search/fabric%20textures
[2] https://pixabay.com/service/license-summary

Example 1[3]

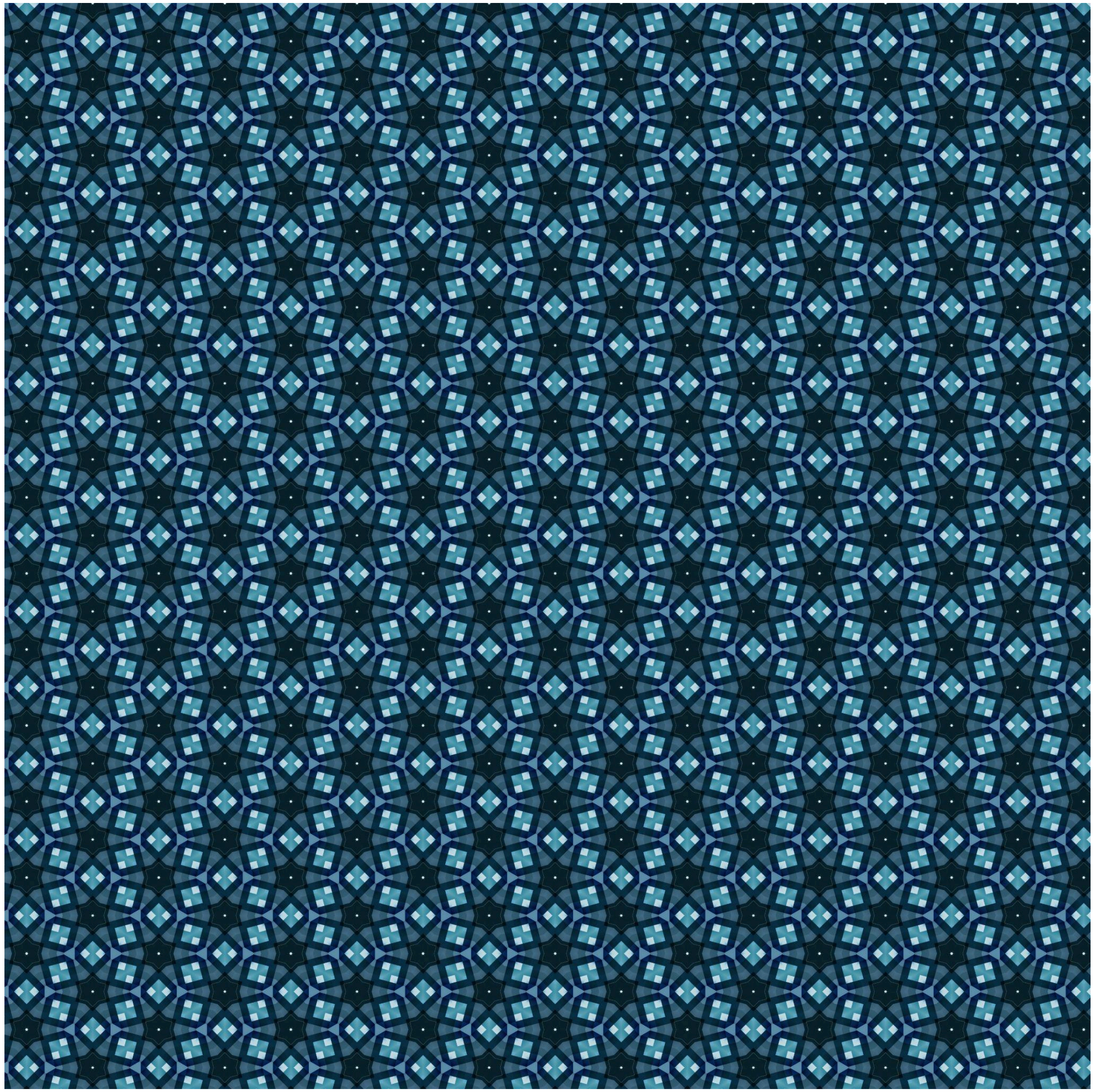

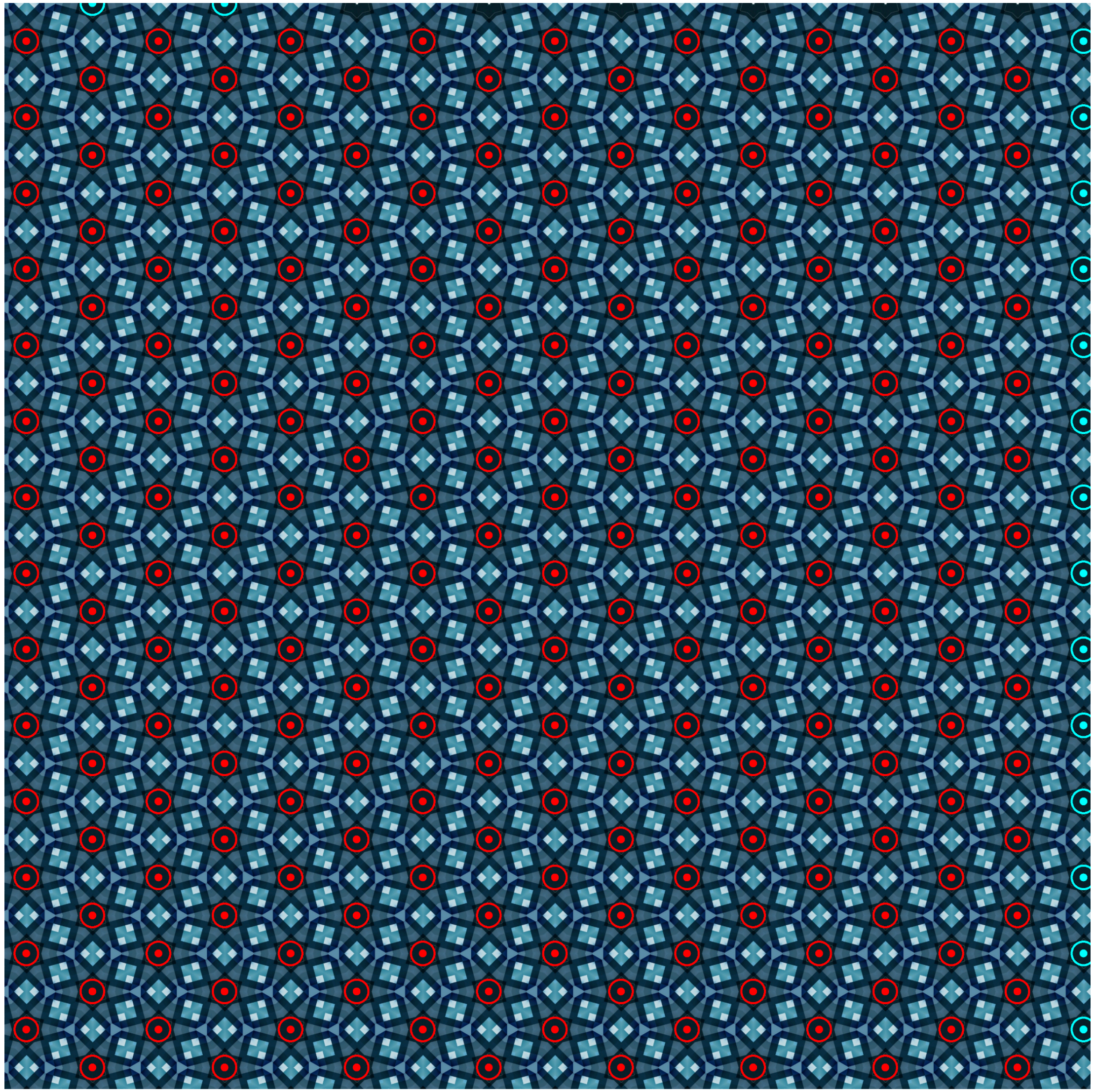

[3] Image source: https://pixabay.com/illustrations/blue-blue-pattern-pattern-design-678472

Example 2[4]

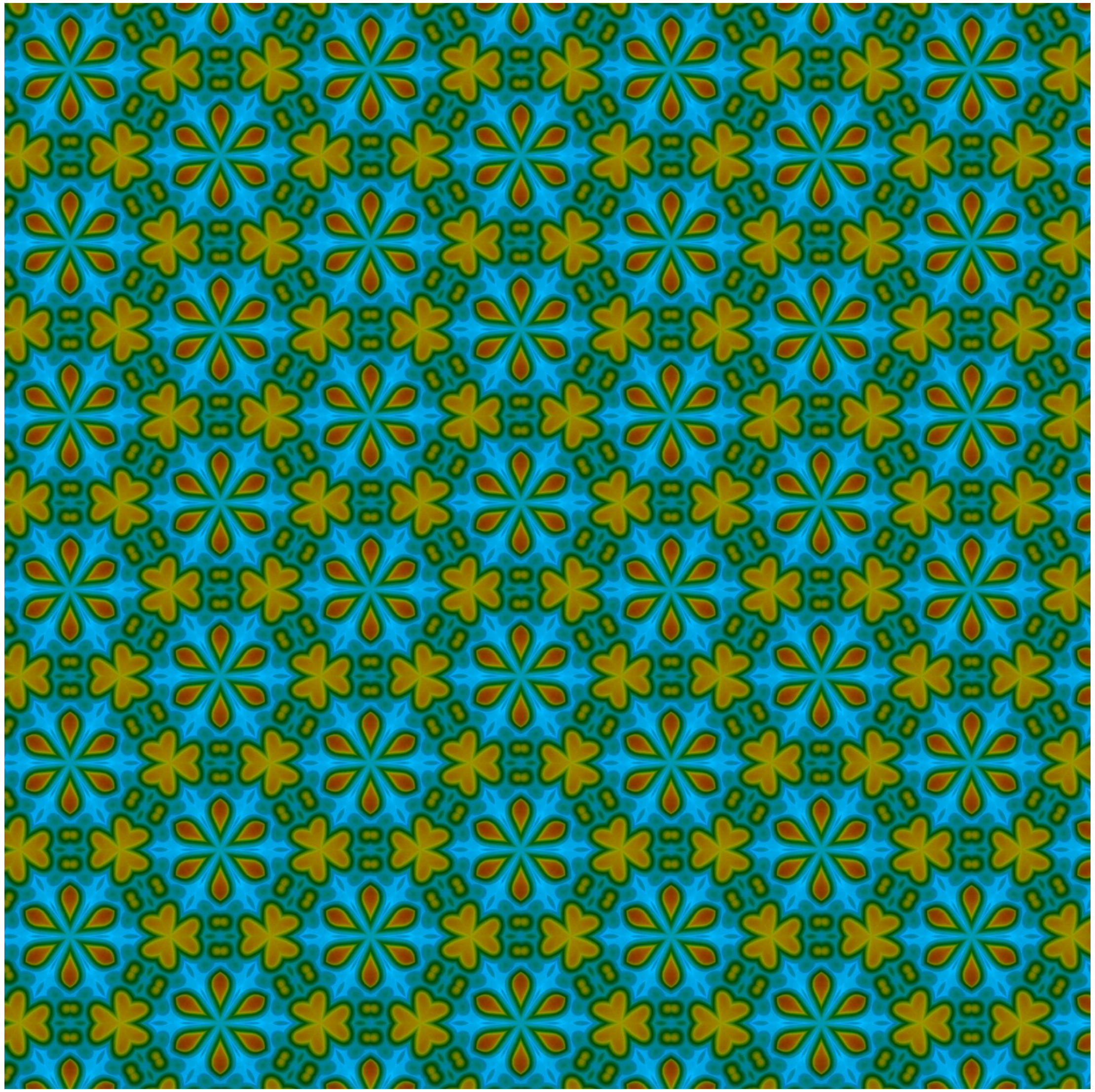

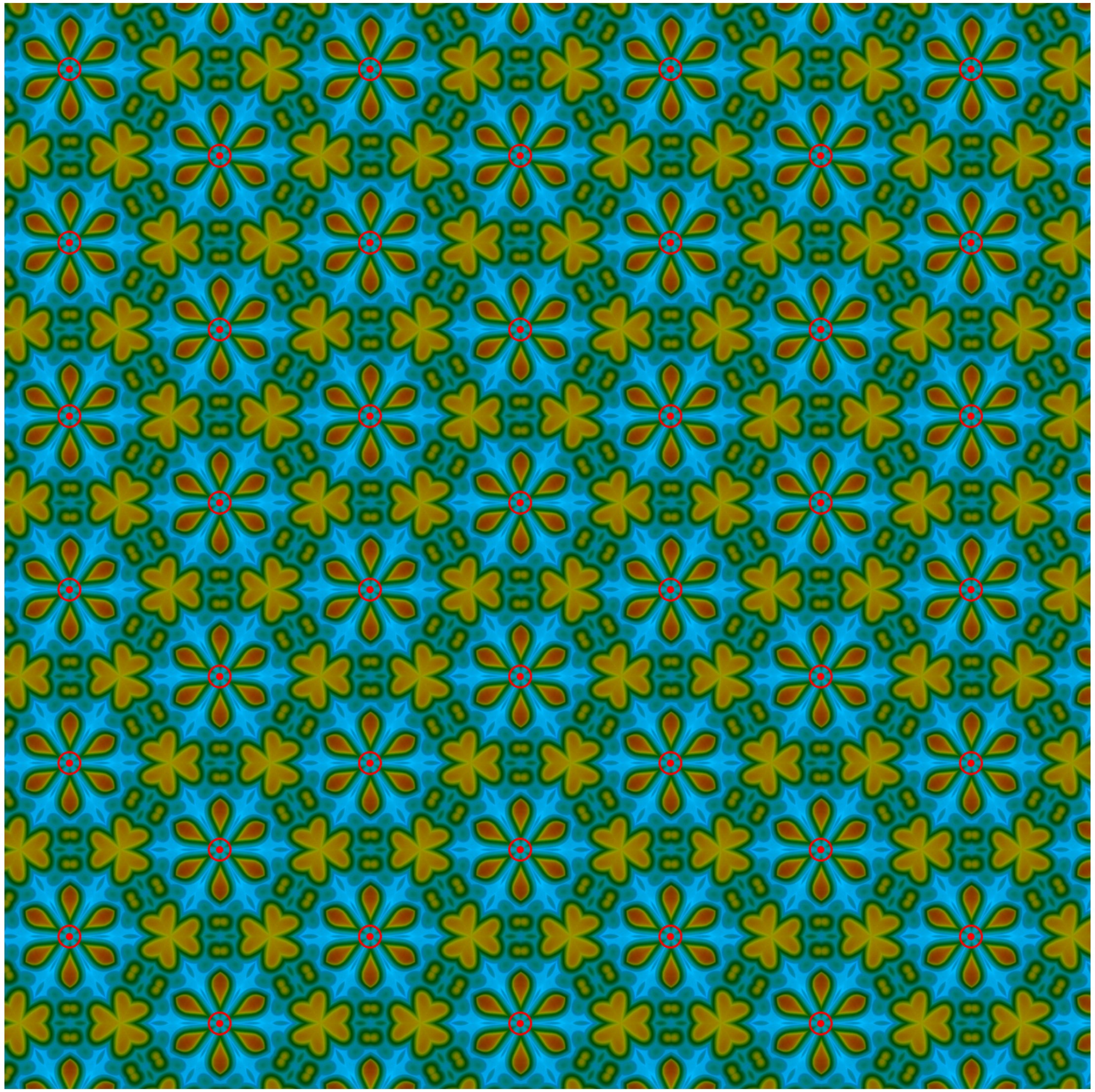

[4] Image source: https://pixabay.com/illustrations/blue-blue-pattern-pattern-design-681556

Example 3[5]

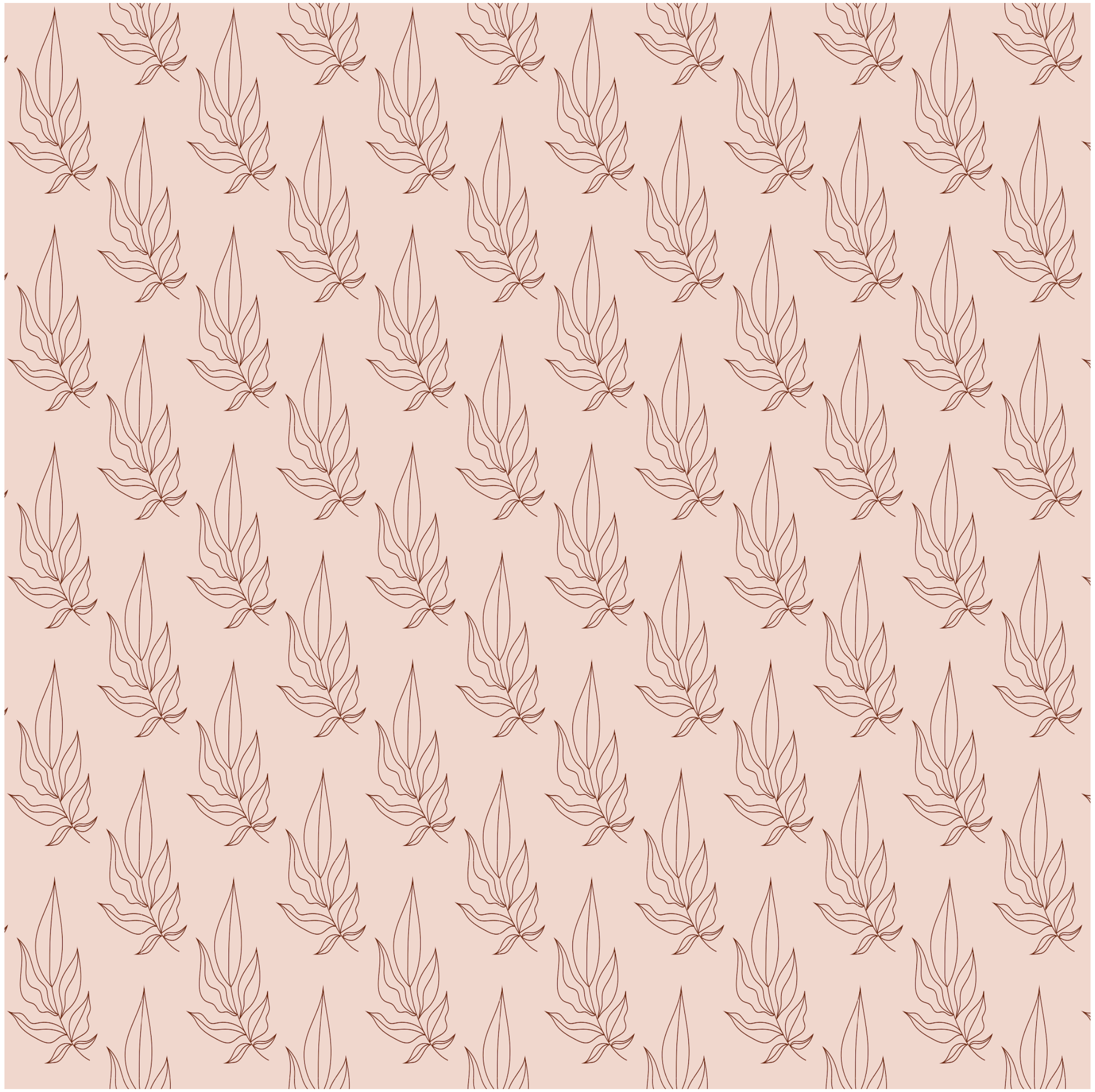

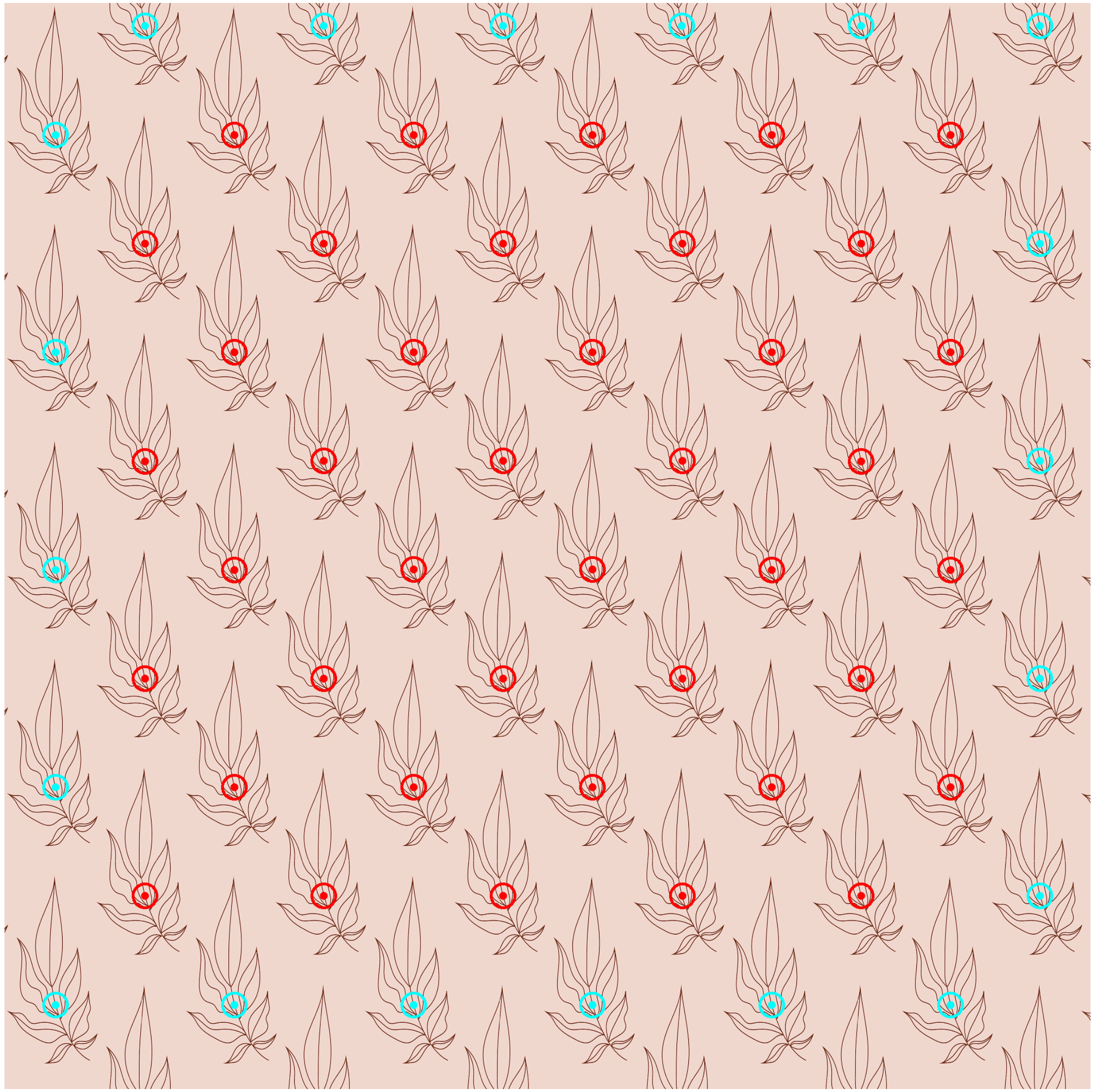

[5] Image source: https://pixabay.com/vectors/pattern-leaves-background-seamless-7448059

Example 4[6]

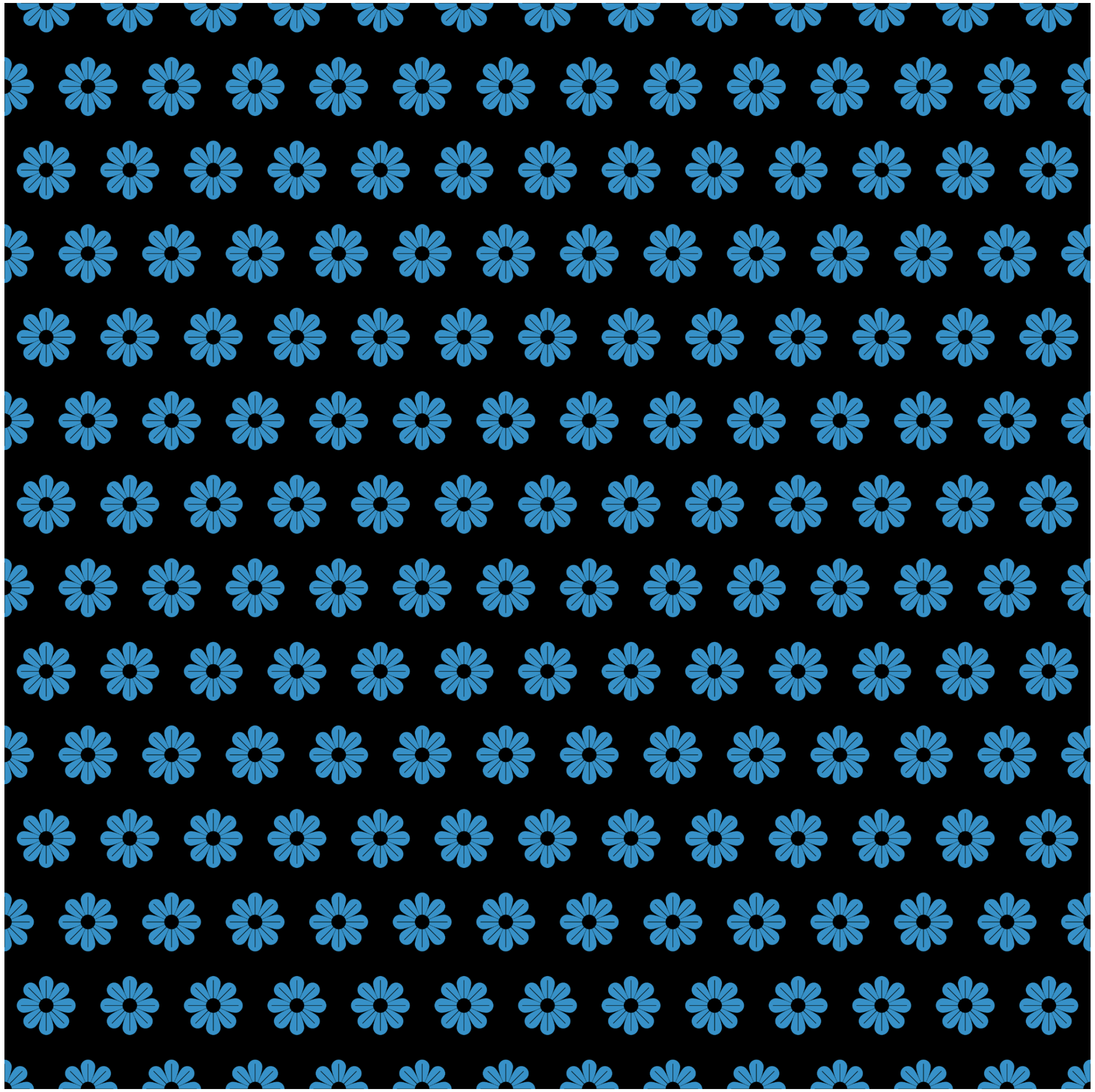

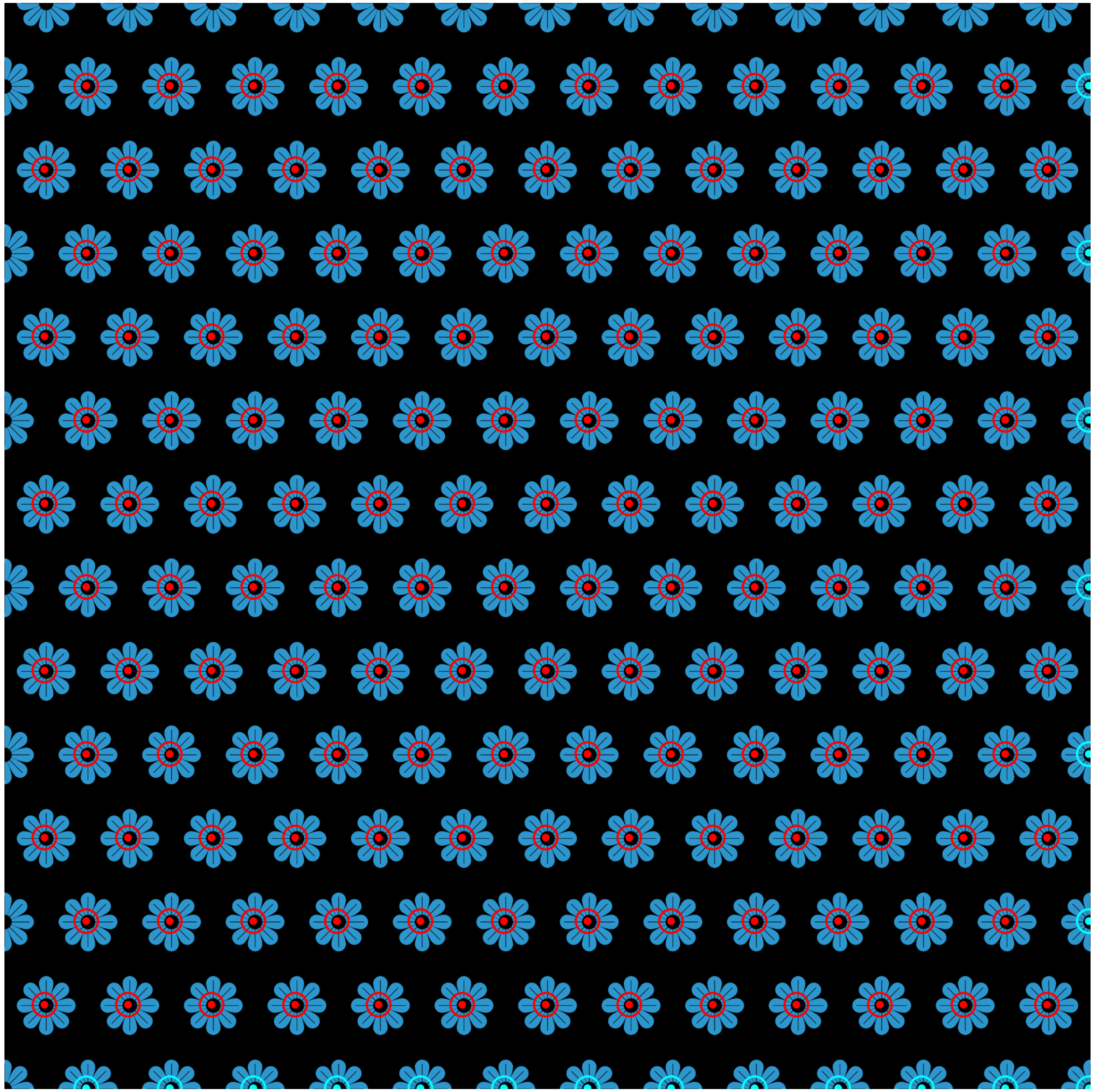

[6] Image source: https://pixabay.com/illustrations/blue-flowers-floral-pattern-7844599

Example 5[7]

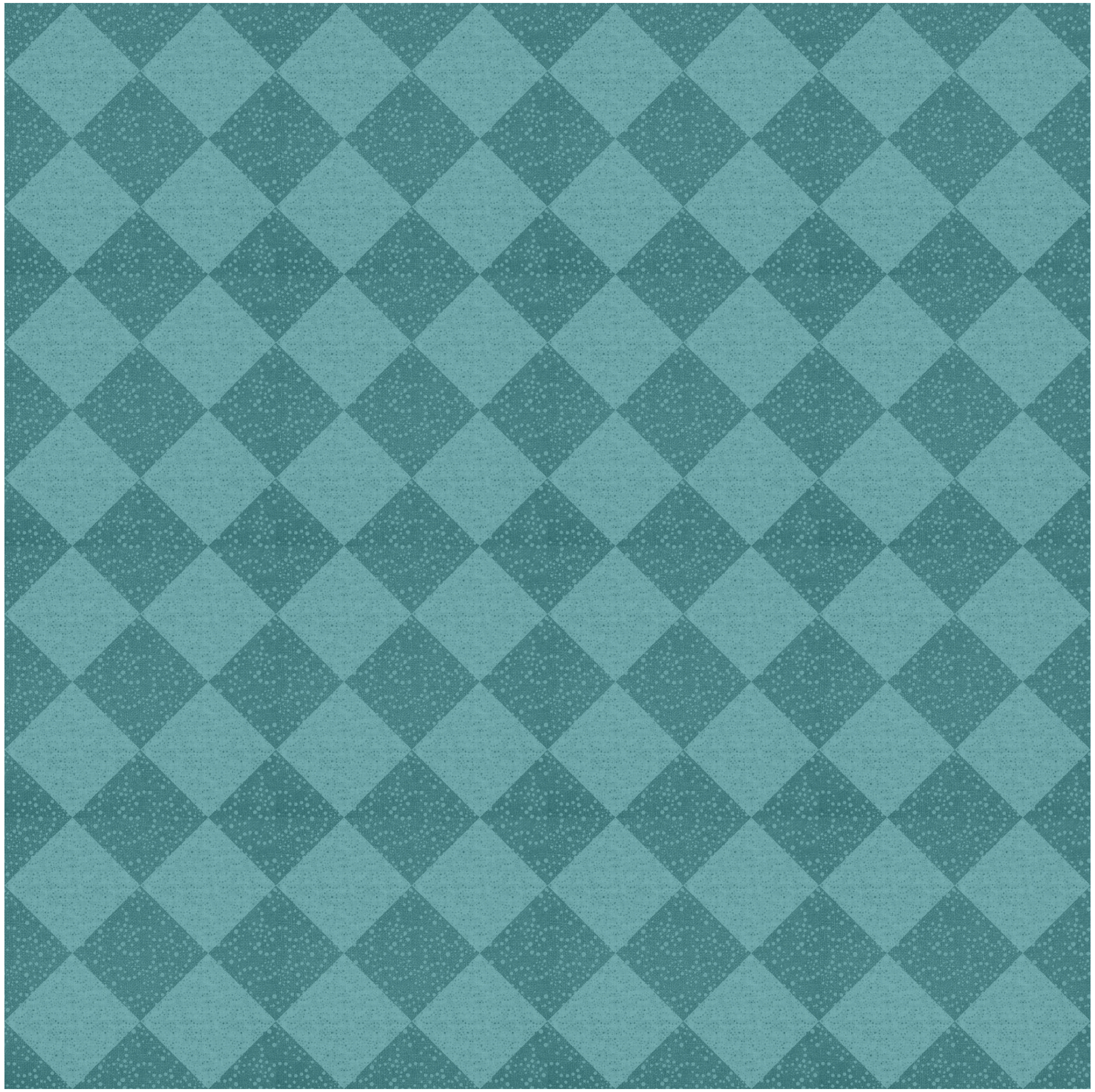

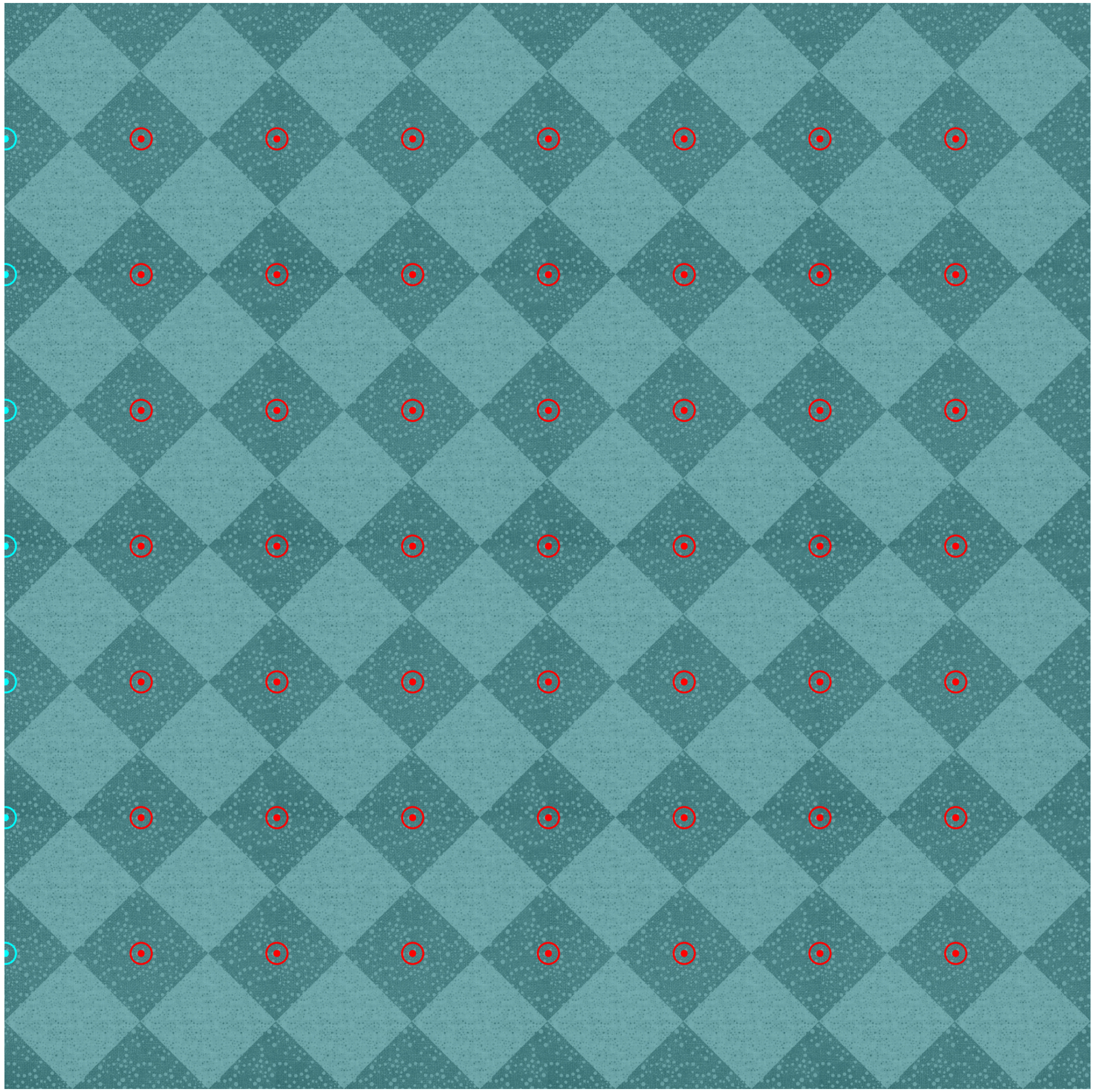

[7] Image source: https://pixabay.com/illustrations/rhomboid-cyan-pattern-background-6281627

Example 6[8]

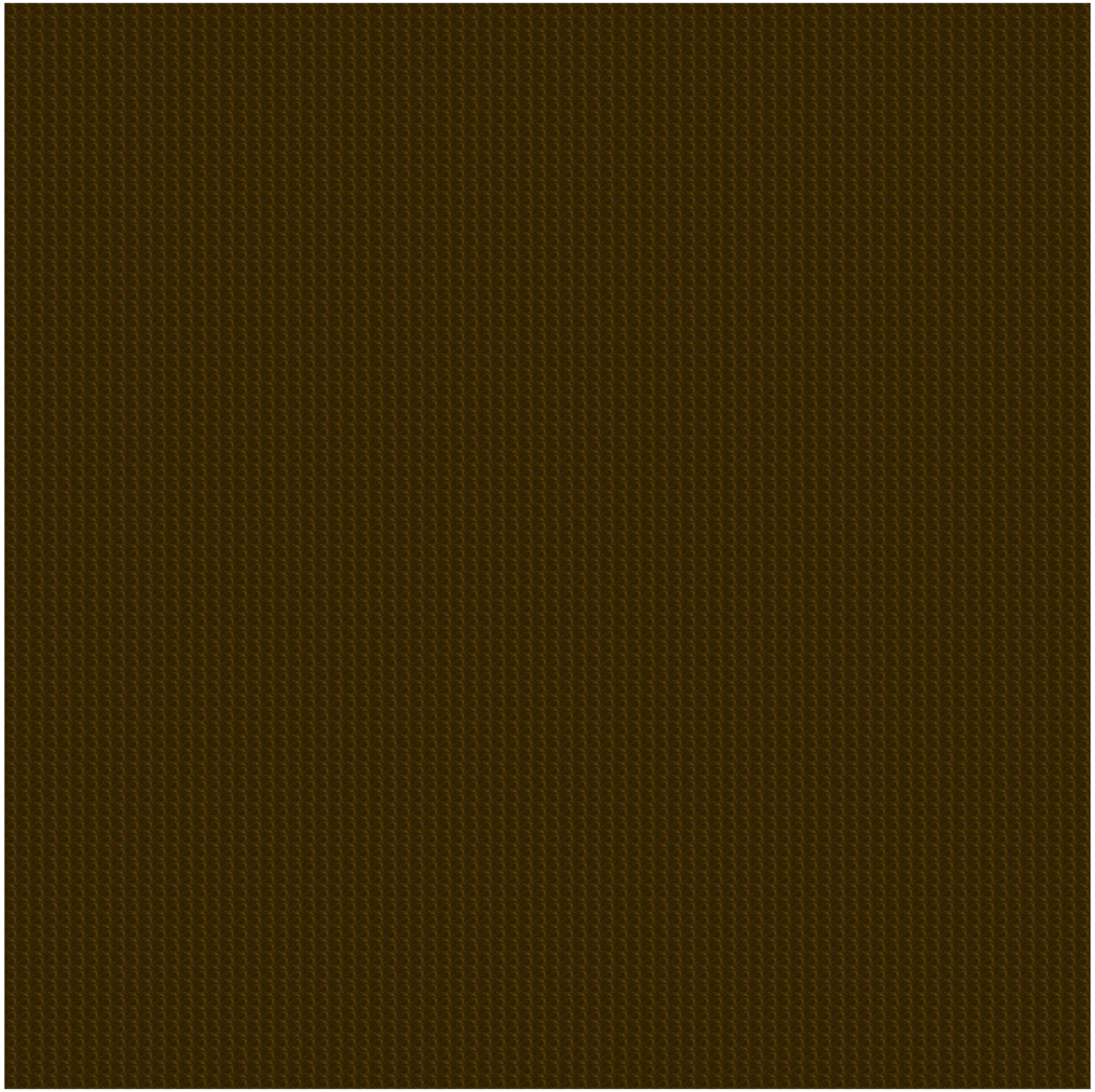

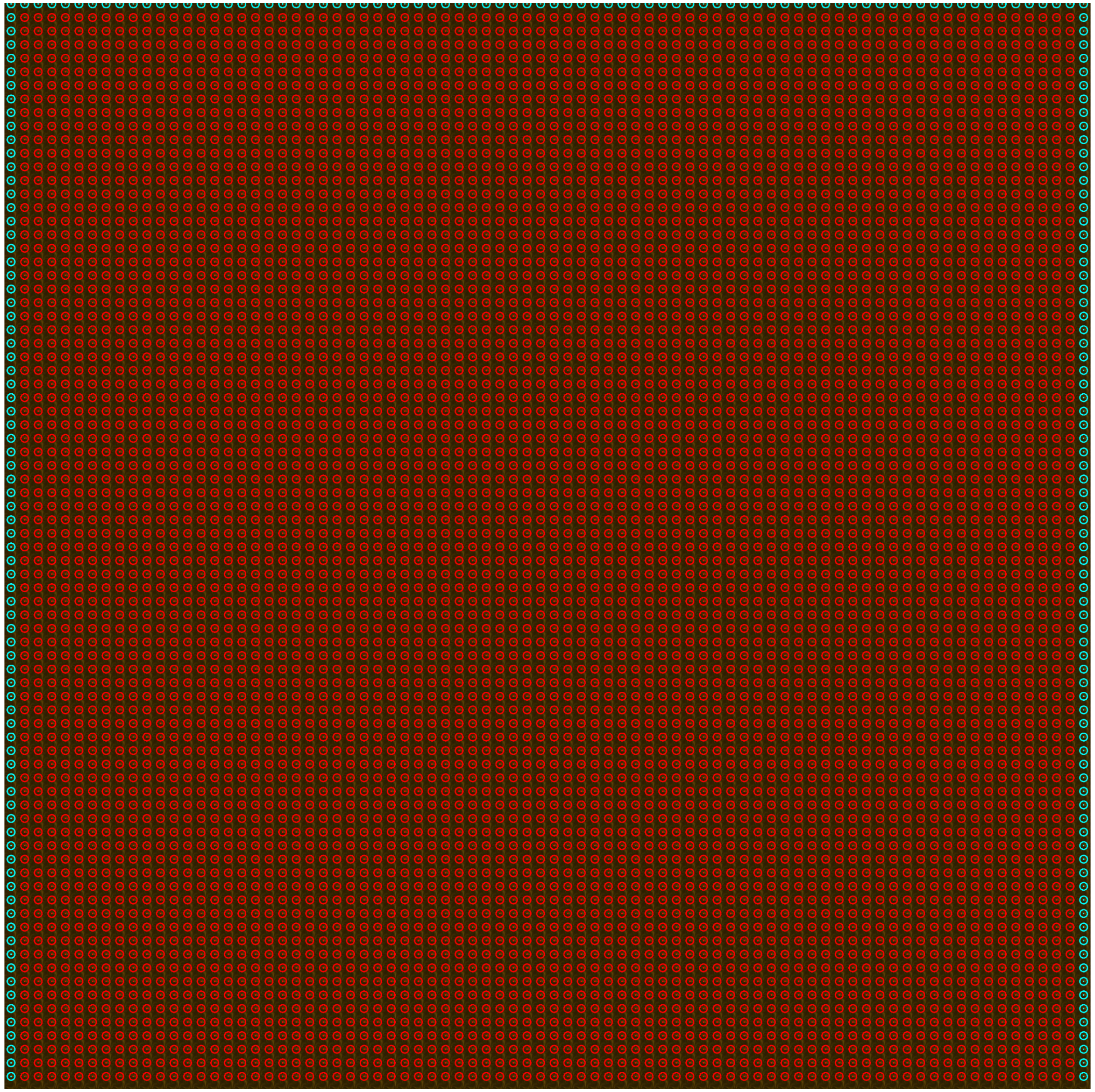

[8] Image source: https://pixabay.com/illustrations/woven-fabric-weave-pattern-texture-8745769

Example 7[9]

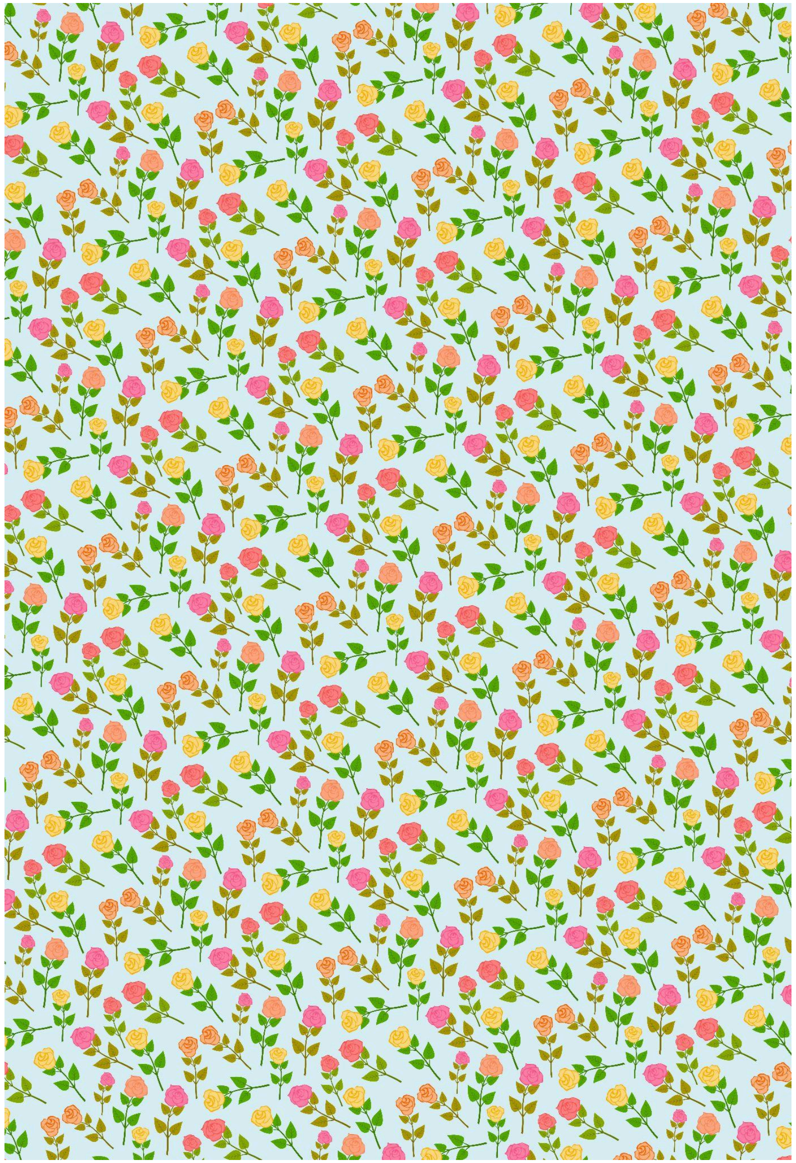

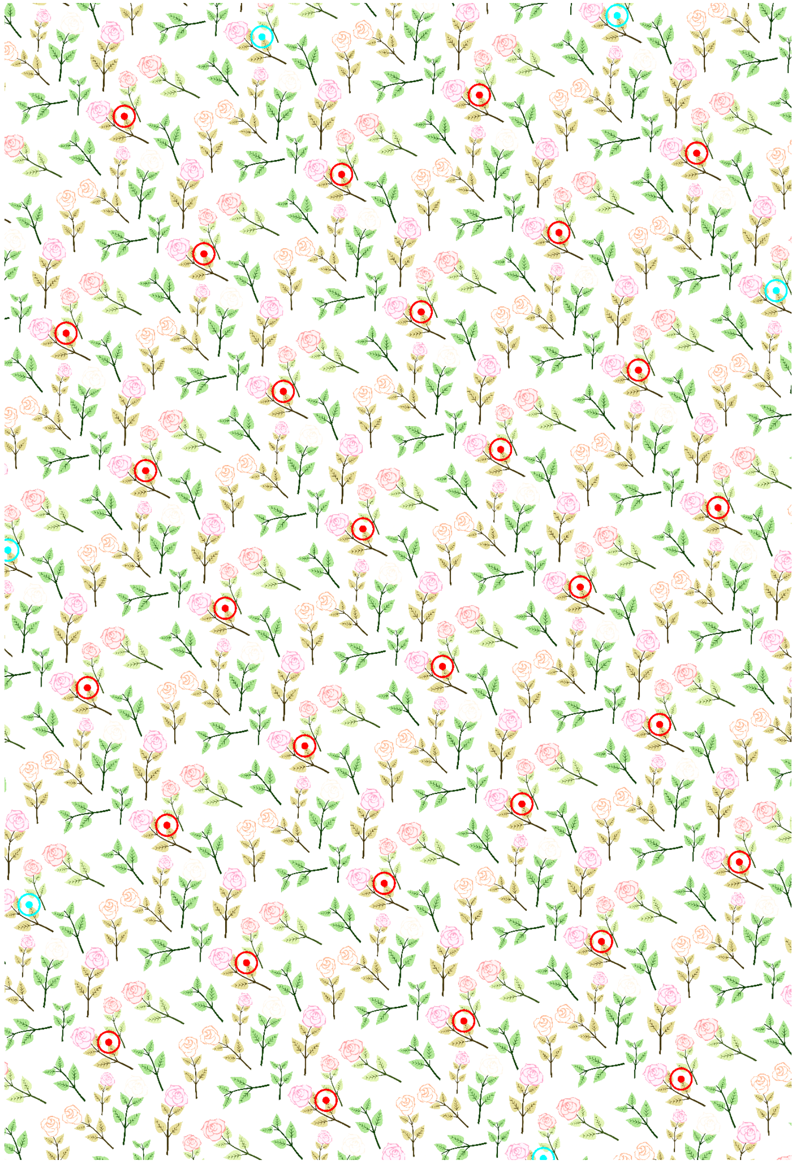

[9] Image source: https://pixabay.com/illustrations/flowers-roses-floral-flowery-2032984

Example 8[10]

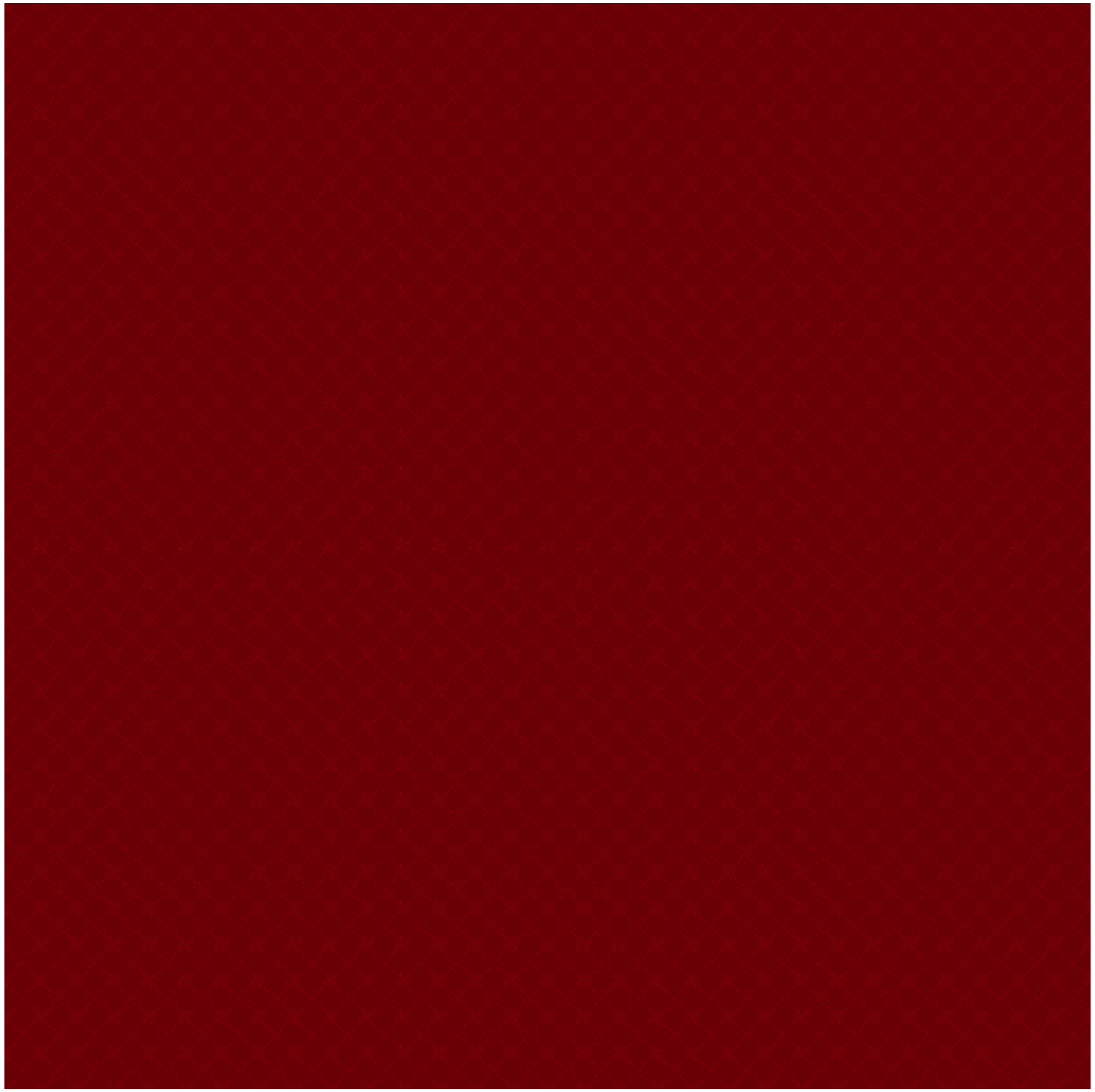

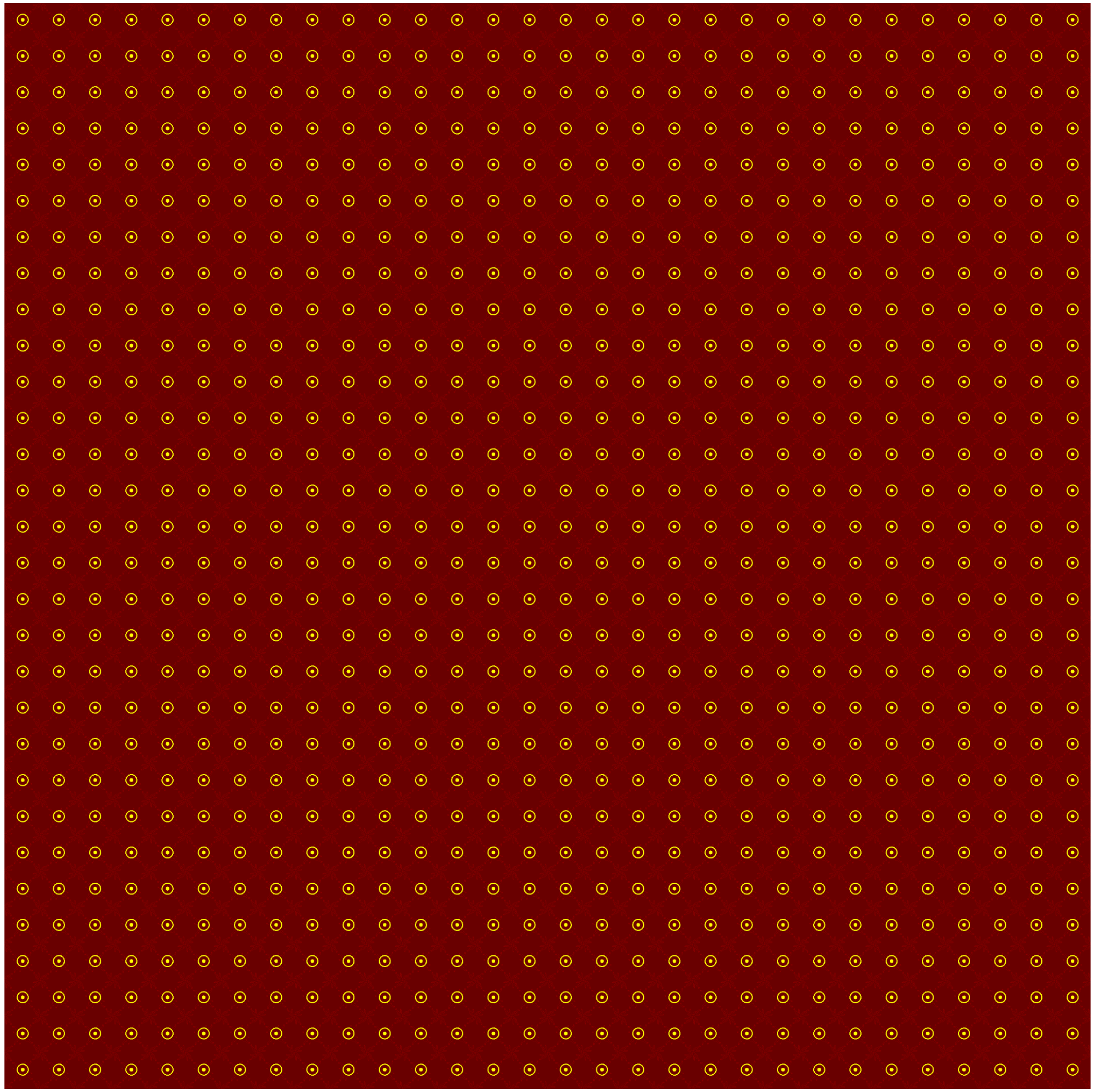

In this example, red circles have been replaced with yellow circles for visibility.

[10] Image source: https://pixabay.com/illustrations/wallpaper-texture-floral-scrapbook-1472852
